# Molecular insight into the specific enzymatic properties of TREX1 revealing the diverse functions in processing RNA and DNA/RNA hybrids

**DOI:** 10.1093/nar/gkad910

**Published:** 2023-10-23

**Authors:** Kuan-Wei Huang, Chia-Yun Wu, Shu-Ing Toh, Tung-Chang Liu, Chun-I Tu, Yin-Hsin Lin, An-Ju Cheng, Ya-Ting Kao, Jhih-Wei Chu, Yu-Yuan Hsiao

**Affiliations:** Department of Biological Science and Technology, National Yang Ming Chiao Tung University, Hsinchu 30068, Taiwan; Institute of Molecular Medicine and Bioengineering, National Yang Ming Chiao Tung University, Hsinchu 30068, Taiwan; Department of Biological Science and Technology, National Yang Ming Chiao Tung University, Hsinchu 30068, Taiwan; Institute of Molecular Medicine and Bioengineering, National Yang Ming Chiao Tung University, Hsinchu 30068, Taiwan; Department of Biological Science and Technology, National Yang Ming Chiao Tung University, Hsinchu 30068, Taiwan; Institute of Molecular Medicine and Bioengineering, National Yang Ming Chiao Tung University, Hsinchu 30068, Taiwan; Department of Biological Science and Technology, National Yang Ming Chiao Tung University, Hsinchu 30068, Taiwan; Institute of Molecular Medicine and Bioengineering, National Yang Ming Chiao Tung University, Hsinchu 30068, Taiwan; Department of Biological Science and Technology, National Yang Ming Chiao Tung University, Hsinchu 30068, Taiwan; Institute of Molecular Medicine and Bioengineering, National Yang Ming Chiao Tung University, Hsinchu 30068, Taiwan; Department of Biological Science and Technology, National Yang Ming Chiao Tung University, Hsinchu 30068, Taiwan; Department of Biological Science and Technology, National Yang Ming Chiao Tung University, Hsinchu 30068, Taiwan; Department of Biological Science and Technology, National Yang Ming Chiao Tung University, Hsinchu 30068, Taiwan; Institute of Molecular Medicine and Bioengineering, National Yang Ming Chiao Tung University, Hsinchu 30068, Taiwan; Institute of Bioinformatics and Systems Biology, National Yang Ming Chiao Tung University, Hsinchu 30068, Taiwan; Center for Intelligent Drug Systems and Smart Bio-devices (IDS^2^B), National Yang Ming Chiao Tung University, Hsinchu 30068, Taiwan; Department of Biological Science and Technology, National Yang Ming Chiao Tung University, Hsinchu 30068, Taiwan; Institute of Molecular Medicine and Bioengineering, National Yang Ming Chiao Tung University, Hsinchu 30068, Taiwan; Institute of Bioinformatics and Systems Biology, National Yang Ming Chiao Tung University, Hsinchu 30068, Taiwan; Center for Intelligent Drug Systems and Smart Bio-devices (IDS^2^B), National Yang Ming Chiao Tung University, Hsinchu 30068, Taiwan; Department of Biological Science and Technology, National Yang Ming Chiao Tung University, Hsinchu 30068, Taiwan; Institute of Molecular Medicine and Bioengineering, National Yang Ming Chiao Tung University, Hsinchu 30068, Taiwan; Institute of Bioinformatics and Systems Biology, National Yang Ming Chiao Tung University, Hsinchu 30068, Taiwan; Center for Intelligent Drug Systems and Smart Bio-devices (IDS^2^B), National Yang Ming Chiao Tung University, Hsinchu 30068, Taiwan; Drug Development and Value Creation Research Center, Center for Cancer Research, Kaohsiung Medical University, Kaohsiung 807378, Taiwan; Department of Biomedical Science and Environmental Biology, Kaohsiung Medical University, Kaohsiung 807378, Taiwan

## Abstract

In various autoimmune diseases, dysfunctional TREX1 (Three prime Repair Exonuclease 1) leads to accumulation of endogenous single-stranded DNA (ssDNA), double-stranded DNA (dsDNA) and DNA/RNA hybrids in the cytoplasm and triggers immune activation through the cGAS–STING pathway. Although inhibition of TREX1 could be a useful strategy for cancer immunotherapy, profiling cellular functions in terms of its potential substrates is a key step. Particularly important is the functionality of processing DNA/RNA hybrids and RNA substrates. The exonuclease activity measurements conducted here establish that TREX1 can digest both ssRNA and DNA/RNA hybrids but not dsRNA. The newly solved structures of TREX1–RNA product and TREX1–nucleotide complexes show that 2′-OH does not impose steric hindrance or specific interactions for the recognition of RNA. Through all-atom molecular dynamics simulations, we illustrate that the 2′-OH-mediated intra-chain hydrogen bonding in RNA would affect the binding with TREX1 and thereby reduce the exonuclease activity. This notion of higher conformational rigidity in RNA leading TREX1 to exhibit weaker catalytic cleavage is further validated by the binding affinity measurements with various synthetic DNA–RNA junctions. The results of this work thus provide new insights into the mechanism by which TREX1 processes RNA and DNA/RNA hybrids and contribute to the molecular-level understanding of the complex cellular functions of TREX1 as an exonuclease.

## Graphical Abstract

**Graphical Abstract:**
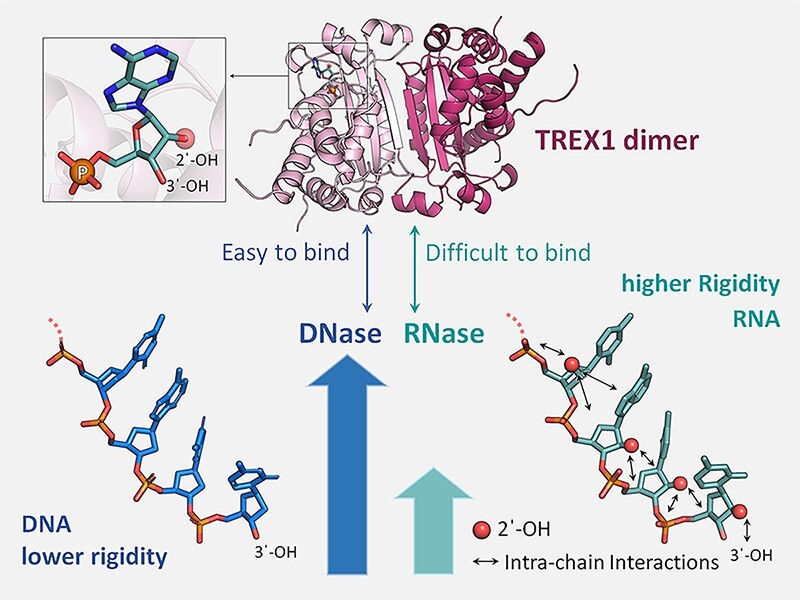

## Introduction

Three prime repair exonuclease 1 (TREX1) is a crucial enzyme in mammalian cells, consisting of an N-terminal nuclease domain and a C-terminal transmembrane domain. TREX1 acts as a 3′-to-5′ exonuclease in the cytoplasm and the nucleus, and has multiple roles in cells (1–3). Under normal conditions, TREX1 is anchored at the endoplasmic reticulum (ER) membrane, where it degrades endogenous DNA to prevent induction of the nucleic acid-mediated immune response. Dysfunction of TREX1 leads to accumulation of cytosolic nucleic acids and a range of autoimmune diseases, such as Aicardi–Goutières syndrome (AGS), systemic lupus erythematosus (SLE), retinal vasculopathy, cerebral leukodystrophy and familial chilblain lupus (FCL) (4–6). As TREX1 acts as an upstream negative regulator of innate immunity, inhibiting its activity may enhance the response of innate immunity, and holds potential for use in cancer immunotherapy (7,8). Furthermore, the nuclease activity of cytosolic TREX1 is linked to the antiviral response, particularly against RNA viruses (9,10).

On the other hand, TREX1 is known to play a role in DNA repair under genotoxic stress conditions, such as UV radiation and benzo(*a*)pyrene exposure. In the presence of such stress, TREX1 is overexpressed and translocated to the nucleus, where it participates in various DNA repair pathways by regulating poly(ADP-ribose) polymerase 1 (PARP-1) (11) or by directly targeting the damaged DNA (3,12–15). The functions of regulating cytosolic immunity and participating in nuclear DNA repair are both highly linked to the complex exonuclease activity and substrate selection mechanisms of TREX1. Thus, it is crucial to understand the catalytic properties of TREX1 exonuclease activity at an atomic level in order to uncover its mysterious cellular functions for targeting TREX1-related diseases. Originally, TREX1 was considered as an exonuclease that specifically targets single-stranded DNA (ssDNA) (12,16). However, later studies showed that TREX1 can also process double-stranded DNA (dsDNA) (3,17,18), and the unwinding of the double-stranded structure is regulated by its Leu24–Pro25–Ser26 (LPS) cluster, its working partner (HMGB-2) and the protein concentration (3). Until now, many questions about the properties of TREX1’s exonuclease activity remain unanswered, such as whether TREX1 is also an RNase and whether it plays a role in processing DNA/RNA hybrids, an essential intermediate in regulating innate immunity and DNA repair.

TREX1 is a member of the DEDDh exonuclease family and adopts a general two-metal ion mechanism for catalysis similar to other members (3,19,20). The DEDDh family is named after the four catalytic residues (DEDD; Asp, Glu, Asp and Asp) and the general base (h; His) for the nuclease reaction (20). This family includes thousands of members across a wide range of species from bacteria to humans, some of which possess both DNase and RNase activities in the metabolic processes of DNA and RNA. For example, the *Escherichia coli* homolog of TREX1, RNase T, plays important roles in DNA repair as well as in RNA maturation (21,22). The TREX1 structures complexed with ssDNA (12,23,24), dsDNA (3,17,18) and deoxyribonucleotides such as dTMP (24,25) and dAMP (26) presented an atomic basis for understanding the DNA catalytic mechanism and provided valuable insight into the molecular origin of the diseases caused by TREX1 mutations. The RNase activity of TREX1, however, has only been confirmed through *in vitro* activity assays (27). Lacking quantitative measurement regarding TREX1–RNA and TREX1–ribonucleotide binding and structural information on TREX1–RNA and TREX1–ribonucleotide complexes makes it difficult to understand how TREX1 binds and processes RNA substrates in the presence of 2′-OH on ribose. The preference for TREX1 in terms of substrate sequence and structures is particularly important to reveal its cellular functions related to RNA metabolism. Furthermore, TREX1 and RNase T exhibit distinct activities in digesting DNA and RNA substrates, and the mechanisms by which they distinguish substrates would provide key insights toward understanding the specific catalytic properties of DEDDh exonucleases. Interestingly, although TREX1 and RNase T are DEDDh exonucleases that both can process DNA as well as RNA (21), they display distinct forms of dimerization structures (28), and the residues stacking the substrate are different (29). A better structural understanding of such functional properties would shed light on the physiological function of TREX1 in processing RNA substrates and establish a framework for analyzing DEDDh exonucleases in terms of DNase and RNase activities.

Cytosolic TREX1 acts to degrade both ssDNA and dsDNA in the cytoplasm to prevent DNA buildup, which triggers the cGAS–STING DNA sensing pathway of type-I interferon (IFN) production and immune system activation (13). Dysfunctional TREX1 was also observed in the immune system activation of autoimmune diseases through the accumulation of DNA/RNA hybrids (30), i.e. they are potentially TREX1 substrates. In cells infected with human immunodeficiency virus (HIV), TREX1 was shown to break down the non-productive HIV reverse transcripts, such as ssDNA and dsDNA, to prevent detection by cytosolic nucleic acid sensors, to suppress IFN production and to avoid the activation of antiviral innate immune responses (9,31–33). Furthermore, in Trex1^−/−^ mouse embryonic fibroblasts (MEFs), the accumulation of HIV-1 DNA/RNA hybrids and IFN production were similarly observed (9), suggesting the functional role of TREX1 in digesting non-productive HIV reverse transcripts in the form of DNA/RNA hybrids. In addition, *in vitro* nuclease activity assays indicated that TREX1 can process the DNA strand as well as the RNA strand in a DNA/RNA hybrid (27). These findings suggest that DNA/RNA hybrids could be natural substrates of TREX1. However, a quantitative understanding of TREX1 in processing ssDNA, ssRNA, dsDNA, dsRNA and DNA/RNA hybrids is lacking. To uncover the complex regulation of innate immunity and DNA repair, it is also crucial to compare the activity of TREX1 with that of other enzymes, such as RNase H2, that can process DNA/RNA hybrids.

In this study, we investigate the activity of TREX1 against various types of nucleic acids, including DNA, RNA, DNA/RNA hybrids and specially synthesized DNA–RNA junctions. The binding affinity of TREX1 for these substrates is measured using quantitative methods. Furthermore, we determine the first structure of a TREX1–RNA product complex and uncover new structures of TREX1 in complexing ribonucleotides as well as deoxyribonucleotides. Together, the study reveals previously unavailable structural information regarding the effect of 2′-OH on exonuclease cleavage. Our results show that RNA and DNA/RNA hybrids are substrates of TREX1, although the activity of digestion is lower compared with that of processing ssDNA. A combination of the structural analysis with molecular dynamics (MD) simulation reveals that the 2′-OH in RNA modulates the conformational rigidity through intra-chain hydrogen bonding, leading TREX1 to exhibit distinctive catalytic properties compared with the processing of DNA. Our results also indicate that the rigidity of RNA through 2′-OH-mediated intra-chain hydrogen bonding may be a key property in the catalysis of other DEDDh exonucleases, such as RNase T, to distinguish DNA and RNA substrates, and could be useful in understanding the functionalities of other nucleic acid-binding proteins. Our establishment of TREX1 digesting RNA without sequence preference is in contrast to the behavior of RNase T. The cellular cooperation of TREX1 with HMGB-2 or RNase H2 in processing various substrates is also discussed. These findings provide new insights into the mechanism of TREX1 in processing RNA and DNA/RNA hybrids and lay the foundation for a better understanding of the complex cellular functions of TREX1 as an exonuclease.

## Materials and methods

### Protein expression and purification

Full-length (encoding amino acids 1–314) and truncated (encoding amino acids 1–242 and 11–242; only the nuclease domain) *trex1* genes of *Mus musculus* (UniProt:Q91XB0) were inserted into the NdeI/XhoI or BamHI/XhoI site of the pET28a vector. The full-length and truncated mTREX1 were employed in biochemical assays, while the truncated (amino acids 1–242) and the N-terminal flexible region-removed mTREX1 (amino acids 11–242) were used for crystallization. The *E*. strain BL21-CodonPlus (DE3)-RIPL (Stratagene, USA) transformed with the expression plasmids were incubated in Luria–Bertani (LB) medium with 50 μg/ml kanamycin, 35 μg/ml chloramphenicol and 25 μg/ml streptomycin. When the OD_600_ of the cell cultures reached ∼0.6–0.7, 1 mM isopropyl-β-d-thiogalactopyranoside (IPTG) was added to induce protein expression at 18°C for 20 h. The harvested cells were disrupted by a high-pressure cell homogenizer (NanoLyzer N2, Gogene Corp. Taiwan) in 50 mM Tris–HCl pH 8.0 containing 300 mM NaCl. The crude protein was centrifuged at 4°C, 13 000 rpm for 30 min to separate the supernatant. TREX1 was further purified from the supernatant sequentially by a HiTrap TALON crude affinity column (Cytiva), a HiTrap Heparin HP column (Cytiva) and a HiLoad 16/60 Superdex 200 prep grade column (Cytiva). The purified TREX1 in 50 mM Tris–HCl, pH 7.0, and 300 mM NaCl was concentrated to ∼28 mg/ml and stored at –20°C. Three recombinant mTREX1 proteins were purified by the same procedures. The TREX1 mutants (H195A and L24A) were generated by QuickChange site-directed mutagenesis kits (Stratagene) and purified by the same procedures as for wild-type mTREX1.

### Nuclease activity assays

The mTREX1 proteins were mixed with various 5′-FAM-labeled nucleic acids (0.5 μM) at 37°C for 30 min in 120 mM NaCl, 20 mM Tris pH 7.0 and 2 mM MgCl_2_. The sequences of substrates are listed in Supplementary Table S1. After adding 2×TBE-urea sample dye and boiling at 95°C for 5 min, the mixtures were subjected to 20% TBE-urea denaturing polyacrylamide gel electrophoresis (PAGE). The FAM-labeled nucleic acid digestion patterns were detected by a blue light-emitting diode (LED) and an electron-multiplying CCD (EMCCD).

### Intrinsic tryptophan fluorescence (ITF)

A 30 μl aiquot of 40 μM protein was mixed into a 270 μl solution containing ligand, 120 mM NaCl and 20 mM Tris–HCl at pH 7.0 with or without 2 mM MgCl_2_. The reaction was allowed to proceed at room temperature for 10 min. For the samples of guanosine or its derivatives, the solution contained 10% Dimethyl sulfoxide (DMSO). All samples were excited at 270 nm. The emission spectrum was measured with 1 nm bandwidth resolution at 290 and 500 nm wavelengths by FluoroMax Plus (Horiba Instruments Inc.) with a 1 ml quartz cuvette. Three measurements were taken for each sample and averaged. The fluorescence intensity collected at 350 ± 3 nm was used for analysis. To verify that the aromatic rings of nucleotides would not interfere with ITF signals, a concentration of 4096 μM of nucleotides was tested under identical experimental conditions without protein (Supplementary Figure S6). The spectra and the fluorescence intensity at 350 ± 3 nm from three independent repetitions were employed for analysis using the GraphPad Prism 8 software. The dissociation constant (*K*_d_) was derived from data by the one-site binding (hyperbola) model: *Y* = *B*_max_ × *X*/(*K*_d_ + *X*), with *X* being the ligand concentration and *B*_max_ being the maximum number of binding sites.

### Crystallization and structure determination

His-tagged truncated mTREX1 (amino acids 1–242 and 11–242) was concentrated at 24–36 mg/ml for crystallization. Protein and input DNA, RNA or nucleotide were mixed for 10 min before crystallization by the vapor diffusion method. The nucleic acid sequence and crystallization conditions are listed in Supplementary Table S2. All data were collected at BL13B1 and BL15A1 in NSRRC, Taiwan, or at the BL44XU at SPring-8, Japan. The data were processed by HKL2000 and determined by the molecular replacement method using Balbes of online-CCP4. The models were refined by Phenix and built by Coot. All statistics of data collection and refinement are listed in Table 1.

**Table 1.** Data collection and refinement statistics (molecular replacement)

	mTREX1–RNA product complex (AMP)	mTREX1–DNA product complex (dNMP)	mTREX1–CMP complex	mTREX1–UMP complex	mTREX1–dAMP complex	mTREX1–uridine complex
Nucleotide/ Nucleoside	AMP	dCMP/dGMP	CMP	UMP	dAMP	Uridine
PDB ID	8HCC	8HCD	8HCE	8HCF	8HCG	8HCH
**Data collection**						
Space group	P12_1_1	P12_1_1	P2_1_2_1_2_1_	P2_1_2_1_2_1_	P3_2_21	P12_1_1
Cell dimensions						
*a*, *b*, *c* (Å)	71.197, 86.433, 75.218	67.680, 81.284, 93.209	64.041, 85.655, 100.034	64.031, 85.771, 100.035	92.585, 92.585, 67.440	67.632, 81.149, 93.653
α, β, γ (°)	90, 97.598, 90	90, 103.688, 90	90, 90, 90	90, 90, 90	90, 90, 120	90, 103.514, 90
Resolution (Å)	30.00–2.00 (2.07–2.00)	30.00–2.00 (2.07–2.00)	30.00–1.50 (1.53–1.50)	30.00–1.60 (1.66–1.60)	30.00–1.80 (1.86–1.80)	30.00–2.00 (2.03–2.00)
*R*_sym_ or *R*_merge_ (%)	7.4 (47.7)	7.3 (46.8)	4.1 (47.6)	5.3 (49.0)	7.1 (48.7)	6.3 (49.5)
I / σ I	25.07 (3.37)	23.32 (2.66)	36.77 (2.51)	46.13 (3.34)	44.91 (3.43)	27.14 (2.20)
Completeness (%)	99.9 (99.9)	99.6 (99.3)	96.2 (95.6)	99.9 (100.0)	99.9 (99.9)	99.6 (99.6)
Redundancy	4.1 (4.0)	4.4 (3.9)	4.5 (4.2)	7.6 (7.3)	9.2 (7.8)	4.3 (3.9)
CC1/2	NA (0.797)	NA	NA (0.766)	NA (0.895)	NA (0.866)	NA (0.725)
CC*	NA (0.942)	NA	NA (0.931)	NA (0.972)	NA (0.964)	NA (0.917)
**Refinement**						
Resolution (Å)	27.16–2.0	28.8–2.0	25.23–1.5	22.83–1.6	27.64–1.8	24.69–2.0
No. of reflections	60 984	65 997	85 156	73 185	31 315	66 276
*R*_work_/*R*_free_	16.72/20.97	19.51/24.03	15.72/19.04	15.29/17.95	16.58/19.33	18.69/22.76
R.m.s. deviations						
Bond lengths (Å)	0.009	0.017	0.01	0.013	0.012	0.004
Bond angles (°)	1.03	1.35	1.08	1.27	1.14	0.68
Ramachandran plot statistics (%)						
Favored region	98.88	98.07	99.55	99.55	99.53	98.83
Allowed region	1.12	1.93	0.45	0.45	0.47	1.17
Outlier region	0	0	0	0	0	0
B-factor (Å^2^)						
All	32.98	37.77	29.76	29.29	40.26	42.34
Protein	32.53	37.19	26.55	27.16	38.84	42.11
Nucleotide/ nucleoside	36.26	40.78	53.82	52.46	62.51	46.06
Water	37.38	42.6	45.36	40.39	47.3	45.58

Each structure was obtained from a single crystal.

Values in parentheses are for the highest resolution shell.

The overall CC1/2 and CC* values were not reported by the version of HKL2000.

The dataset of 8HCD was processed in 2012 with no CC1/2 and CC* values reported by the version of HKL2000.

### All-atom MD simulation

The all-atom models of TREX1 bound with ssDNA and ssRNA were constructed based on the X-ray structure of the TREX1–DNA complex (PDB code: 5YWS) with the positions of missing atoms patched using the CHARMM software (34). The last six DNA residues in 5YWS were retained as the substrate sequence (GCCATC) in the TREX1–DNA simulation, and the (GCCAUC) substrate sequence was used in the TREX1–RNA simulation. The CHARMM36 protein (35) and nucleic acid (36) all-atom force fields were employed to calculate molecular interactions in the potential energy function, while the GROMACS software (Abraham *et al.* GROMACS 2023 Manual. 2023; https://doi.org/10.5281/zenodo.7588711.) was used to conduct MD simulations. Both TREX1–DNA and TREX1–RNA systems were solvated in a dodecahedron box of TIP3P water with at least 1.0 nm between any protein atom and the periodic boundary, and counter ions at 1.4 mM ionic strength. The cut-off of van der Waals (VDW) and short-range electrostatic interactions was 1.2 nm with a switching function effective at 1.0 nm, and the long-range electrostatics were calculated by Fast smooth Particle-Mesh Ewald with a grid spacing of 0.1 nm. During the all-atom MD simulations, all covalent bonds associated with a hydrogen atom were constrained at the equilibrium distance. After 4 ns of heating and equilibration, the production run was conducted at 310 K and 1.103 bar for 2 μs. The sampled configurations were saved every 10 ps for analysis. To determine the statistical uncertainties of hydrogen bonding strengths, the trajectory was equally divided into four independent blocks by using a longer duration between the snapshots.

### Fluorescence anisotropy measurements

The serial 2-fold dilutions of mTREX1 (amino acids 1–242) were separately mixed with FAM-labeled nucleic acids (MDBio, Inc.) into 300 μl aliquots. These mixtures containing 5 nM nucleic acid and different concentrations of mTREX1 were equilibrated at room temperature for 30 min in 120 mM NaCl, 20 mM Tris pH 7.0 and 20 mM EDTA. The fluorescence signals were measured by a fluorescence spectrometer, FluoroMax Plus (Horiba Instruments Inc.), equipped with a linear polarizer (TECHSPEC High Contrast Plastic Linear Polarizers, Edmund Optics). The fluorescence anisotropy, *r*, was calculated as *r* = (*I*_VV_*I*_HH_)/(*I*_VH_*I*_HV_ + 2 *I*_VH_*I*_H_), with *I* being the fluorescence signal intensity of FAM and subscripts H and V indicating the horizontal and vertical orientation of the excitation and emission polarizers, respectively. Each fluorescence signal of FAM was the average over 5 s with 494 nm excitation (1 nm slit) and 520 nm emission (5 nm slit). All three independent repeat data were analyzed using the GraphPad Prism 8 software. The fluorescence anisotropy was plotted as a function of the mTREX1 concentration. These binding curves were fitted into the software built-in non-linear regression fitting mode, *Y* = *B*_max_ × *X*/(*K*_d_ + *X*) + Background × *K*_d_/(*K*_d_ + *X*), to attain the dissociation constant *K*_d_ values. Here, *B*_max_ is the maximum fluorescence anisotropy signal of the saturation binding curve and Background is the basic fluorescence anisotropy signal of free nucleic acids.

## Results

### TREX1 processes ssRNA and the RNA strand in DNA/RNA hybrids

**Figure 1.**
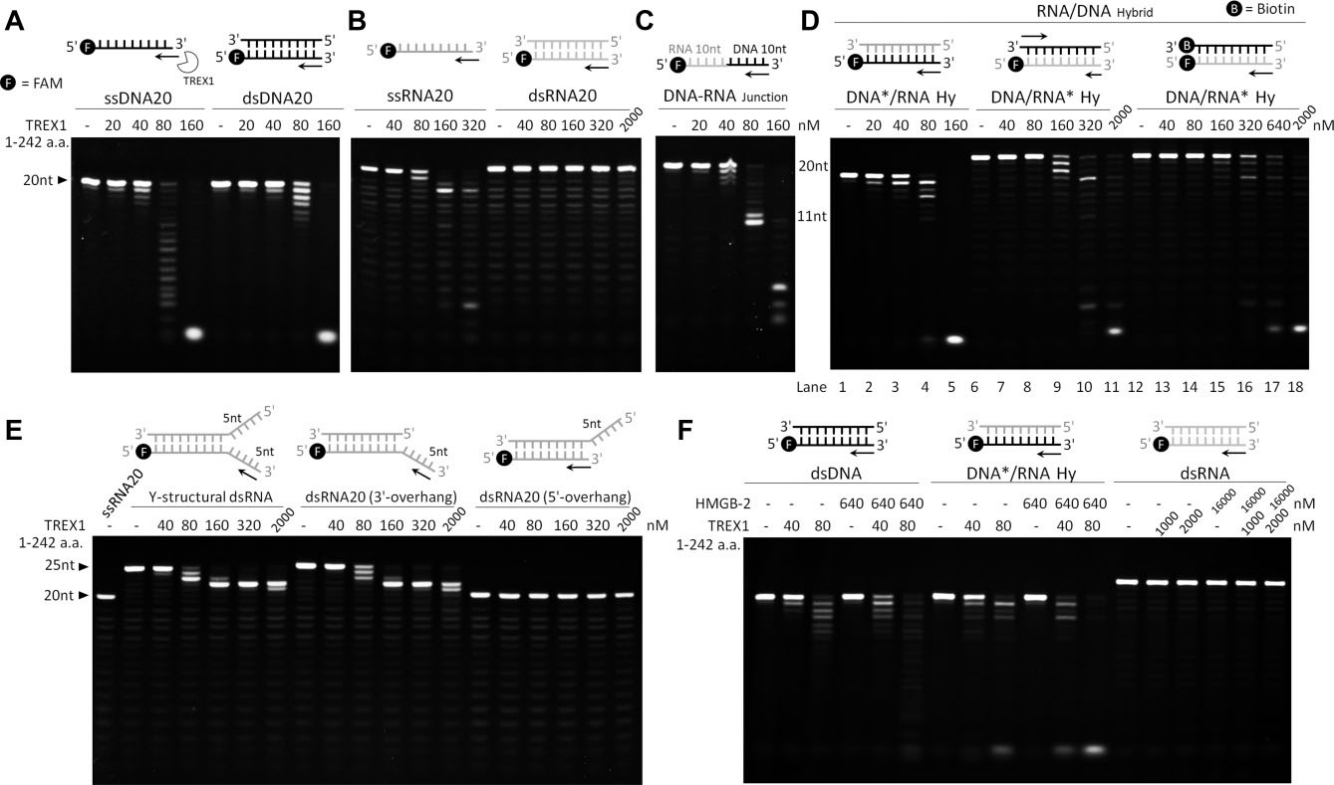
Catalytic properties of truncated and full-length TREX1 on various DNA and RNA substrates. (**A**–**D**) Nuclease activity assays of truncated mTREX1 (nuclease domain only, amino acids 1–242) on digesting ssDNA (20 mer), dsDNA (20 bp), ssRNA (20 mer), dsRNA (20 bp), DNA/RNA hybrid (20 bp) and the DNA–RNA junction (20 mer). The concentrations of substrates used in the reactions are 0.5 μM. The black circle with the letter F represents FAM. The black and gray strands represent DNA and RNA strands, respectively. (**E**) Nuclease activity assays of truncated mTREX1 on digesting duplex RNA with a 5 nt long 5′ overhang or a 5 nt long 3′ overhang and Y-shaped. The double-strand structure of RNA blocks mTREX1’s activity, even when the concentration of mTREX1 reaches 2000 nM. (**F**) Assays of TREX1 exonuclease activity in the presence or absence of mouse HMGB-2 with various double-stranded substrates. TREX1 cleaves dsDNA and DNA/RNA hybrid more efficiently in the presence of HMGB-2 but still cannot digest dsRNA.

To understand the molecular basis of TREX1 in processing RNA and DNA/RNA hybrids, mouse TREX1 (mTREX1) is expressed and purified for biochemical and structural analysis. The length and sequence of mTREX1 closely resemble the those of human TREX1 isoform b more than isoforms a and c (27), and the domain structure is shown in Supplementary Figure S1A. The His-tagged wild-type (amino acids 1–314), truncated and mutated mTREX1 expressed in *E. coli* were purified to homogeneity through chromatographic methods, as confirmed by sodium dodecylsulfate (SDS) electrophoresis (Supplementary Figure S1A, B). The exonuclease activity of transmembrane domain-truncated mTREX1 (amino acids 1–242, consisting only of the nuclease domain) was examined by incubation with FAM-labeled ssDNA, dsDNA and DNA/RNA hybrids. The result shows that mTREX1 digests ssDNA with higher activity and processes the DNA strand in DNA/RNA hybrids with similar rates to that in the degradation of dsDNA (Figure 1A, D).

Truncated mTREX1 displays a higher activity in digesting ssRNA than cutting the RNA strand in DNA/RNA hybrids, and no activity is observed in processing dsRNA (Figure 1B, D). This result shows that mTREX1 also functions as an RNase with an activity approximately half that of its DNase role. This property is established based on the early digestion patterns of ssDNA and ssRNA substrates at concentrations of 40 and 80 nM, respectively (Figure 1A, B). The different levels of mTREX1 activity in processing DNA and RNA can also be seen in the accumulation of bands when using an RNA–DNA junction as the substrate (Figure 1C). Full-length mTREX1 (amino acids 1–314) and truncated mTREX1 exhibit similar catalytic properties in processing the RNA substrates (Supplementary Figure S1D). The active site mutant, truncated mTREX1 H195A, demonstrates very limited activity in breaking down various DNA and RNA substrates, thereby supporting that the exonuclease activity observed in the nuclease activity assays originates from mTREX1 (Supplementary Figure S2A).

In targeting DNA/RNA hybrids, mTREX1 displays a higher activity in digesting the DNA strand than in degrading the RNA strand (Figure 1D). To better assess the strand-specific activity of TREX1 toward DNA/RNA hybrids, the 3′ end of the DNA strand is labeled with biotin to prevent excessive digestion and thereby expose the RNA strand as ssRNA. The results show that mTREX1 can digest the RNA strand in DNA/RNA hybrids but with a lower activity than the degradation of ssRNA and the DNA strand in DNA/RNA hybrids (Figure 1D).

The most notable difference between the DNase and RNase activity of mTREX1 is that even at 2 μM mTREX1, i.e. 25 times the necessary concentration for digesting ssRNA, dsRNA remains intact (Figure 1B). On the other hand, only 2-fold the enzyme concentration for ssDNA digestion is required for TREX1 to process dsDNA or DNA/RNA hybrids (Figure 1A, D). Using Y-structural duplex RNA or duplex RNA with 3′ or 5′ overhangs as substrates, mTREX1 can remove the single-stranded 3′-overhang region but not the double-stranded parts (Figure 1E). Regarding the processing of double-stranded substrates, previous studies showed that the presence of a cellular working partner HMGB-2 can elevate the activity of TREX1 (3). To determine the impact of HMGB-2 on the digestion of DNA/RNA hybrids and dsRNA, mouse HMGB-2 (mHMGB-2; Supplementary Materials and methods; Supplementary Figure S1B) is purified and included in the nuclease activity assay. The results show that the addition of mHMGB-2 indeed raises the catalytic efficiency of TREX1 against dsDNA and DNA/RNA hybrids but does not affect the inability to cut dsRNA (Figure 1F).

To better assess the RNase activity of mTREX1 in comparison with other RNases, such as RNase H1 and RNase H2, mouse RNase H1 and H2 (mRNase H1 and H2) are expressed and purified into homogeneous states (Supplementary Materials and methods; Supplementary Figure S1C). The result shows that the RNA strand in DNA/RNA hybrids begins to break down at mRNase H1 and H2 concentrations of 80 and 40 nM, respectively (Supplementary Figure S2B). For reference, the required concentration of mTREX1 for ssRNA digestion is 80 nM. These *in vitro* experiments indicate that the RNase activity levels of these three mouse nucleases are within a similar range.

### Crystal structures of mTREX1 complexed with DNA or RNA products

To reveal the previously unavailable structural information on the mechanism by which the 2′-OH affects RNA processing, the crystal structures of mTREX1 in complexation with DNA or RNA products were determined. With the various RNA or DNA substrates digested by mTREX1 during crystallization, only a single nucleotide is left in the active site. Two structures of mTREX1 complexed with the DNA or RNA product are obtained at 2.0 Å resolution. The AMP and dGMP in our structures of the TREX1–RNA product complex and the TREX1–DNA product complex align well with the 3′-ended deoxyribonucleotides observed in the previously solved structures of TREX1–DNA substrate complexes [including ssDNA (PDB code: 2OA8) (26) and dsDNA (PDB code: 5YWS) (3); Supplementary Figure S3]. This analysis also indicates an RNA or DNA product-bound form of TREX1 in our newly solved structures. For comparison, additional structures of mTREX1–deoxyribonucleotide or mTREX1–ribonucleotide complexes were determined at 1.5–1.8 Å resolution. The crystallization conditions, data collection and refinement statistics of these five structures are shown in Supplementary Table S2 and Table 1. The F_o_–F_c_ omit maps for the various nucleotides in these structures reported in Supplementary Figure S3B display a clear and precise fit with the structures of the nucleotides.

**Figure 2.**
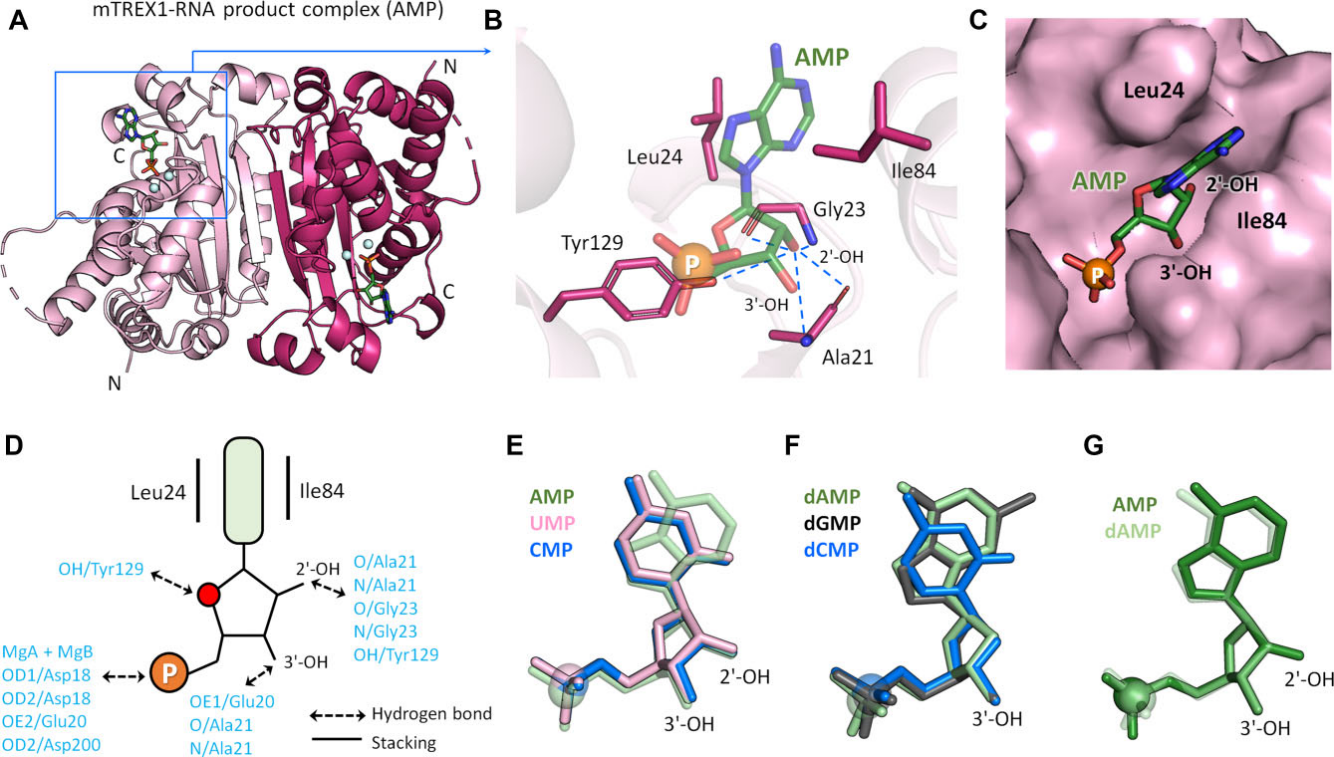
Overall and active site structures of mTREX1–RNA product and mTREX1–DNA product complexes. (**A**) The overall structure of mTREX1 in complex with the RNA product, adenosine monophosphate (AMP). The two monomers of the TREX1 dimer are colored pink and warm pink, respectively. The light blue balls are Mg ions. (**B**) A close up view of the active site of the mTREX1–RNA product complex. Hydrogen bonds are shown as blue dotted lines. (**C**) The surface structure of the active site of the mTREX1–RNA product complex. (**D**) Schematic representation of the interactions between mTREX1 and AMP. (**E**–**G**) The superposition of various RNA products (AMP), DNA products (dCMP and dGMP) and nucleotides. The phosphates of nucleotides are displayed as balls.

The structure of the mTREX1–RNA product complex (AMP) showcases a traditional dimeric conformation of mTREX1, with two magnesium ions and AMP filling the active site. The base region of AMP is stacked by Leu24 and Ile84 (Figure 2A, B). To verify the critical role of Leu24 in stacking RNA, the purified L24A mutant of TREX1 is shown to have reduced RNase activity (Supplementary Figure S2C). In comparison with the mTREX1–DNA structure, the extra 2′-OH group in the ribonucleotide interacts with the main chains of Gly23 and Ala21 and the side chain of Tyr129 through hydrogen bonds (Figure 2B, D). The three-dimensional structure of the ribonucleotide with the extra 2′-OH group fits well into the active site of mTREX1, demonstrating hindrance-free binding with the ribonucleotide (Figure 2C).

To understand how substrate binding depends on the base structure, the ribonucleotides in the mTREX1–RNA product (AMP) and mTREX1–ribonucleotide (CMP and UMP) structures as well as the deoxyribonucleotides in the mTREX1–DNA product (dGMP and dCMP) and mTREX1–deoxyribonucleotide (dAMP) structures were superposed. Our analysis reveals that AMP, CMP and UMP all align well, with the scissile phosphate and ribose in identical positions (Figure 2E). Similarly, superimposing the deoxyribonucleotides shows consistent results (Figure 2F). To explore the difference between deoxy- and ribonucleotides, a comparison of the superimposed structures of dAMP and AMP shows a consistent binding position, except for the absence of the 2′-OH group in the former (Figure 2G). In addition, superposition with AMP, dAMP (26) and dTMP (24) deoxyribonucleotides solved in earlier TREX1 structures also shows similar positions (Supplementary Figure S4). Overall, our analyses demonstrate that the binding positions, relative positions and residues interacting with TREX1 are highly similar across various deoxyribonucleotides and ribonucleotides.

### mTREX1 exhibits similar affinity with deoxyribonucleotides and ribonucleotides

Previous studies showed that TREX1 and its homolog TREX2 function as non-processive exonucleases by excising one nucleotide at a time from the 3′ end of a DNA substrate (37–39). Without the transmembrane domain, TREX2 shares >40% sequence identity with TREX1 in the nuclease domain and similar substrate preferences (39). The structures of TREX2 and TREX1 dimers also exhibit homology (Supplementary Figure S5A). The TREX2 active site is a narrow pocket and can host the 3′-end nucleotide product. Due to the closed TREX2 active site with only one entrance, the 3′-end nucleotide of a new substrate cannot enter the active site unless the previously cleaved duplex DNA and nucleotide are released. This structural feature was recognized as the cause of the inability of TREX2 to carry out processive processing of duplex DNA (39). Along the same line, our analysis of the mTREX1–RNA product complex and previous TREX1–substrate complex structures (PDB code: 5YWS) (3) reveals that mTREX1 has a similar active site structure, with the cleaved nucleotide adopting a similar conformation (Supplementary Figure S5). Therefore, TREX1 exhibits non-processive enzyme catalysis as does TREX2.

**Figure 3.**
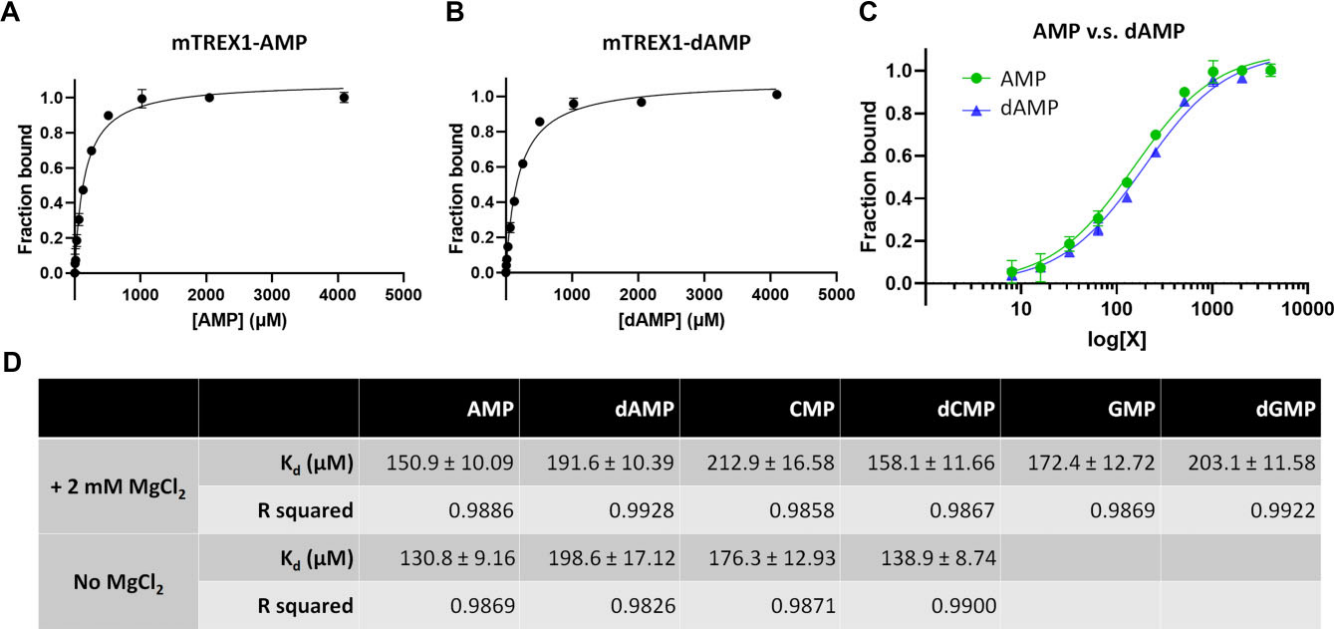
The ITF measurement of the *K*_d_ between mTREX1 and various nucleotides. (**A** and **B**) Quantification of the *K*_d_ between TREX1 and AMP or dAMP in the presence of 2 mM MgCl_2_. The dissociation constants are calculated using a one-site binding model. (**C**) Comparison of the binding between AMP (green) and dAMP (blue). X is the AMP or dAMP concentration. (**D**) The *K*_d_ of TREX1 with various ribonucleotides and deoxyribonucleotides, including AMP, dAMP, CMP, dCMP, GMP and dGMP, in the presence or absence of 2 mM MgCl_2_.

For non-processive exonucleases, the product release rate is critical in determining the catalytic efficiency, and investigation of TREX1 binding with deoxyribonucleotides and ribonucleotides sheds light on the molecular origin of substrate preference. In this regard, intrinsic tryptophan fluorescence (ITF) is employed to measure the binding affinity of mTREX1 for different nucleotides. The aromatic ring of nucleotides is shown not to interfere with the ITF signals (Supplementary Figure S6). The results depicted in Figure 3 and Supplementary Figure S7 indicate that the equilibrium dissociation constant (*K*_d_) of mTREX1 with AMP, dAMP, CMP, dCMP, GMP and dGMP falls between 130 and 210 μM, with no specific difference between deoxyribonucleotides and ribonucleotides. Despite the two-metal ion catalysis (19,20), similar *K*_d_ values are obtained in the absence of Mg^2+^ ions and, under such solution conditions, a difference between binding deoxyribonucleotides and ribonucleotides is also not observed (Figure 3D; Supplementary Figure S8). This finding suggests that the additional 2′-OH group on ribose does not affect binding, and the product release rates of TREX1 in processing DNA and RNA are expected to be similar.

### The mTREX1–uridine structure reveals that 2′-OH does not impose steric hindrance

**Figure 4.**
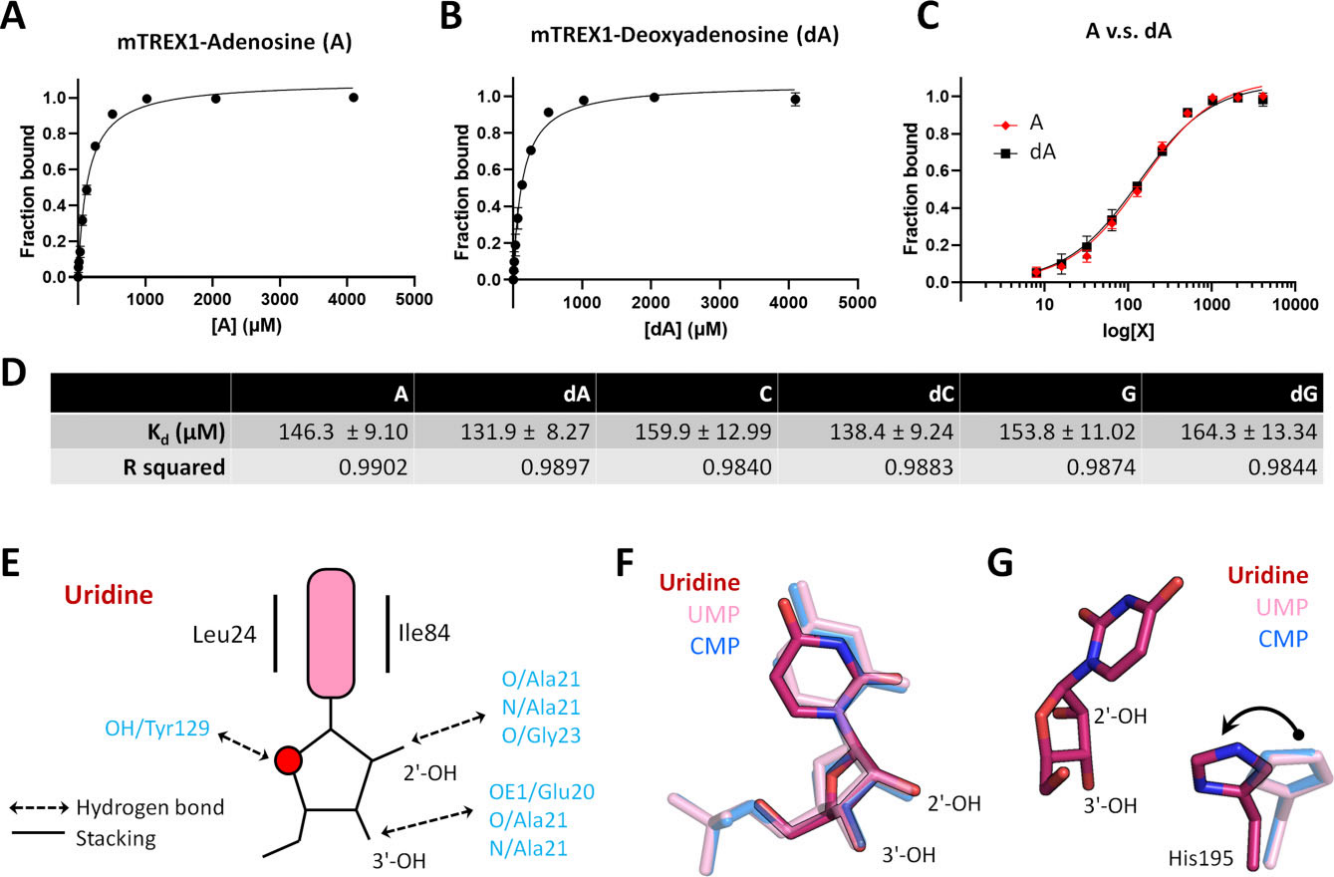
The ITF measurement of *K*_d_ between mTREX1 and various nucleosides. **(A** and **B**) Quantification of the *K*_d_ between TREX1 and adenosine (A) or deoxyadenosine (dA) in the presence of 2 mM MgCl_2_. The dissociation constants are calculated using a one-site binding model. (**C**) Comparison of the binding between A (red) and dA (black). X is the A or dA concentration. (**D**) The *K*_d_ of TREX1 with various nucleosides and deoxynucleosides in the presence or absence of 2 mM MgCl_2_, including A, dA, cytidine (C), deoxycytidine (dC), guanosine (G) and deoxyguanosine (dG). (**E**) Schematic representation of the interactions between mTREX1 and uridine. (**F**) The superposition of CMP, UMP and uridine showing the well-fit structures of nucleotides and nucleosides. (**G**) Superposition of the general base, His195, in the TREX1–uridine, TREX1–UMP and TREX1–CMP complex structures. His195 is closer to uridine in the TREX1–uridine complex structure.

The structure of the mTREX1–uridine complex is determined for further elucidation of the role of 2′-OH, and the *K*_d_ of mTREX1 with various nucleosides and deoxynucleosides is measured using ITF. The results presented in Figure 4 and Supplementary Figure S9 indicate that 2′-OH does not lead to a significantly different affinity for TREX1 to bind nucleosides and deoxynucleosides (Figure 4D). The residues involved in interacting with uridine and 2′-OH are identical to those in nucleotides (Figure 4E). The structural alignment of uridine and UMP also reveals similar positions in the active site of mTREX1, with the main difference being that the general base His195 is closer to uridine (Figure 4F, G).

### Intra-chain hydrogen bonding in RNA affects binding with TREX1

**Figure 5.**
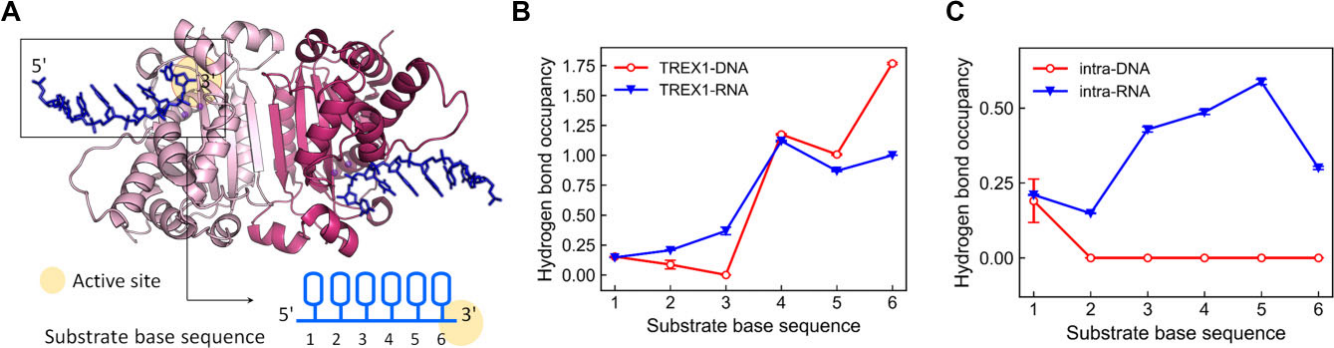
All-atom MD simulation of TREX1–DNA and TREX1–RNA complexes. (**A**) The all-atom model of TREX1–DNA and TREX1–RNA simulations based on the 5YWS structure. The ssDNA or ssRNA substrate contains six base residues with the 3′ end bound to the active site. MD simulations of the two systems were conducted in explicit solvent for 2 μs of the production run. (**B**) The hydrogen bond occupancy averaged over the production run between substrate residues and TREX1. The 1.0 occupancy of the active site magnesium ion with the phosphodiester backbone at the 3′ end is included in base 6. (**C**) The hydrogen bond occupancy averaged over the production run between substrate residues. In both (B) and (C), occupancy values are averaged over those of the two protomers.

The above analysis illustrates that the 2′-OH of the last nucleotide of RNA does not lead to structural hindrance in the active site pocket and has an insignificant effect on the binding affinity and the product release rate with TREX1. What then is the molecular origin of the apparently different catalytic power of TREX1 in digesting ssDNA and ssRNA (Figure 1)? Since the intra-chain interactions mediated by 2′-OH play a key role in leading to the different mechanical properties of DNA and RNA (40), their distinct rigidities could be an important feature in causing the apparently lower efficiency for TREX1 to process RNA substrates. To test this hypothesis, all-atom MD simulations of TREX1 bound with six base long ssDNA or RNA (ssRNA) were conducted in an aqueous environment with explicit solvent (each atom in water is explicated represented in the simulation model) (Figure 5). The analysis of each simulation is based on a 2 μs production run collected at 300 K and 1 atm. As shown in Supplementary Figure S10, the TREX1 structural core hosting DEDD contains three β-strands and two helices, and shows a very small (<1.0 Å) and plateaued C_α_-atom RMSD (root-mean-square difference) values over the trajectory, indicating robustness in the conformation. The 124–140 segment containing the helix of Asp130 and the substrate contacting Tyr129 (cf. Figure 2B), for example, shows a <1.0 Å C_α_-RMSD evaluated after aligning the trajectory snapshots with respect to the core C_α_ positions in the reference X-ray structure (hlx4 in Supplementary Figure S10). Other structural segments around the DNA or RNA substrate, such as the 174–188 segment and the loop containing the His195 general base, on the other hand, exhibit significantly larger positional variation (Supplementary Figure S10, hlx6 and l195). Therefore, the TREX1–DNA and TREX1–RNA interactions are subject to heterogeneous structural flexibilities in the protein dynamics, and could be modulated by specific intra-chain hydrogen bonding in the substrate to affect the binding with TREX1.

Given the structure of a snapshot, the occupancy of a hydrogen bond is counted as 1.0 if the donor–acceptor distance is <3.0 Å and the donor hydrogen–acceptor angle is >150°. A permanent hydrogen bond would thus have an averaged 1.0 occupancy over the trajectory, and the interaction strength between a substrate sequence and TREX1 is the sum of all hydrogen bond occupancies mediated by the DNA or RNA residue. An example of persistent interaction in the simulation of the TREX1 complex is between one of the non-bridging oxygen atoms in the phosphodiester bond of base 6 and one of the active site Mg^2+^ ions. The 1.0 value of this interaction is included in the total occupancy between TREX1 and the 3′-end residue in Figure 5B. The >1.0 occupancy (∼1.75) of base 6 in TREX1–DNA thus indicates the presence of additional hydrogen bonding, whereas the 1.0 value of the base in TREX1–RNA shows that the Mg^2+^–backbone coupling is the dominant 3′-end interaction with the substrate.

Figure 5B indicates that the hydrogen bonding of DNA or RNA with TREX1 primarily occurs at the last three bases at the 3′ end, and the TREX1–DNA occupancy is evidently higher than that of TREX1–RNA. Consistent with the X-ray structures of TREX1, Glu20, Ala21, Tyr129 and His195 contribute significant portions in the occupancy with ssDNA base 6, primarily with backbone phosphate oxygens and ribose O3′ and O4′. Additional details of the protein–DNA interactions toward the 5′ end are discussed in Supplementary Figure S11. Although the overall interaction pattern observed in the TREX1–RNA simulation is similar, the occupancy values are much lower. The statistically significant signals of the TREX1–nucleic acid hydrogen bonding strengths shown in Figure 5B suggest that the TREX1–DNA binding would be stronger than that of TREX1–RNA and is consistent with TREX1 having lower activity in processing ssRNA than that in digesting ssDNA. With 2′-OH, ssRNA indeed exhibits significant intra-chain hydrogen bonding, mostly between 2′-OH and the ribose O4′ of neighboring bases, in the all-atom MD simulation while ssDNA does not (Figure 5C). The RMSF (root-mean-square fluctuation) values of the 3′-end residues that TREX1 interacts with are thus consistently lower in ssRNA than those in ssDNA, (Supplementary Figure S11), indicating the reduced structural flexibility due to the intra-chain hydrogen bonding in the substrate. The higher rigidity in ssRNA thus comes with weaker coupling with TREX1. The analysis of MD simulation shows that although the 2′-OH does not introduce steric hindrance, it mediates higher intra-chain rigidity, and the lower flexibility in the polymer chain would affect TREX1 binding.

### The binding affinity of mTREX1 on various DNA and RNA substrates

**Figure 6.**
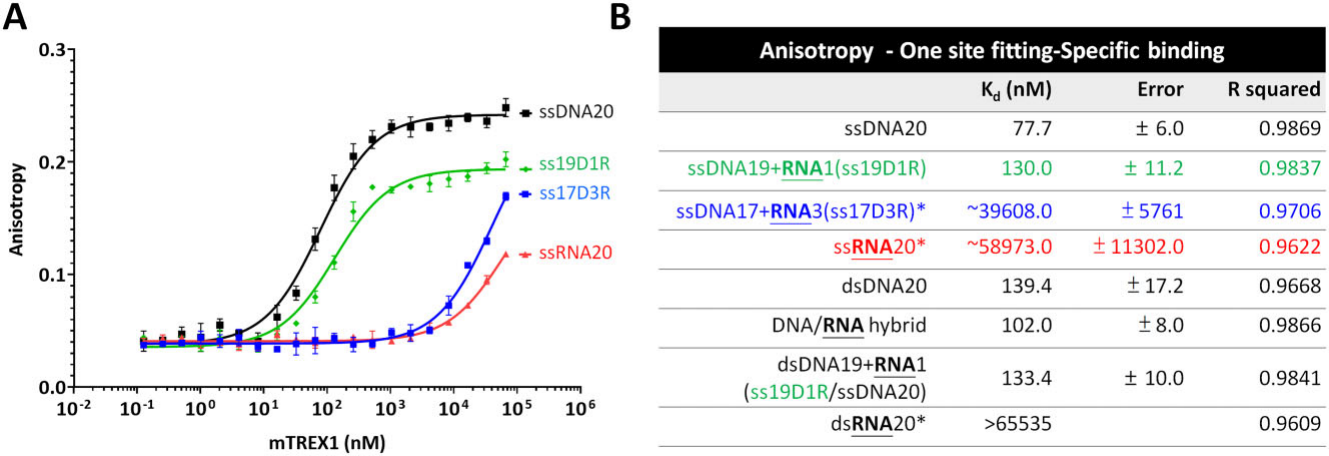
Fluorescence anisotropy measurements of truncated mTREX1 on various substrates. (**A**) Fluorescence anisotropy of ssDNA20 (ssDNA 20 mer), ssRNA20 (ssRNA 20mer), ssDNA19 + RNA1 (synthesized ssDNA–ssRNA junction, including ssDNA 19 mer and one 3′-ended ribonucleotide) and ssDNA17 + RNA3 (synthesized ssDNA–ssRNA junction, including ssDNA 17 mer and 3′-ended ssRNA 3 mer). The substrates labeled with FAM at the 5′ end are plotted as a function of added mTREX1 protein. (**B**) The dissociation constants (*K*_d_) of mTREX1 binding various nucleic acid substrates, including ssDNA20, ssDNA19 + RNA1 (ss19D1R), ssDNA17 + RNA3 (ss17D3R), ssRNA20, dsDNA20 (dsDNA 20 bp), DNA/RNA hybrid (DNA/RNA hybrid 20 bp), dsDNA19 + RNA1 (ss19D1R anneals to ssDNA 20 mer) and dsRNA20 (dsRNA 20 bp). The symbol * indicates that for binding curves that do not reach saturation, *K*_d_ values are estimated by extrapolation, and the *K*_d_ values of ssRNA and ss17D3R are ∼58 973 and 39 608 μM, respectively. The extrapolated *K*_d_ of dsRNA20 is >65 535 nM.

The structural analysis demonstrates that the 2′-OH in RNA does not cause steric hindrance, and all-atom MD simulations indicate that the intra-hydrogen bonds of RNA play a crucial role in the lower activity of TREX1 against RNA substrates. To further test this mechanism, we synthesized FAM-labeled ssDNA and ssRNA for measuring the *K*_d_ of the binding of mTREX1 with DNA, DNA–RNA junction and RNA substrates by fluorescence anisotropy (Figure 6A, B; Supplementary Figure S12).

Consistent with the prediction of all-atom MD simulations, our results show that ssDNA indeed has a higher binding affinity (lower *K*_d_) for mTREX1 than does ssRNA. The *K*_d_ of ssDNA was measured to be 77.7 ± 6.0 nM, but the TREX1–ssRNA binding does not reach saturation, with an estimated *K*_d_ of 58 973.0 ± 11 302.0 nM. To further investigate the impact of 2′-OH of a single ribonucleotide on DNA, a 20 nt long DNA–RNA junction substrate is synthesized (ss19D1R) to have 19 nt long ssDNA and a 3′-end ribonucleotide. The binding affinity of ss19D1R for mTREX1 is reduced by ∼40% compared with the ssDNA 20 mer, indicating that the single 2′-OH at the 3′-ended ribonucleotide is a contributing factor to mTREX1 binding, although the impact is lower compared with that of ssRNA. However, when another 20 nt long DNA–RNA junction substrate is used, which consists of 17 mer ssDNA plus three continuous ribonucleotides at the 3′ end (ss17D3R), the *K*_d_ in the presence of more intra-chain hydrogen bonding decreases dramatically by ∼500-fold compared with the 20 mer ssDNA and is close to the value of binding ssRNA (Figure 6A, B). The result is also consistent with our all-atom MD simulations showing that TRE1–DNA and TREX1–RNA binding primarily couple with the last three residues at the 3′ end. Our study establishes that intra-chain hydrogen bonding in RNA is a critical effector for binding with TREX1.

With double-stranded substrates, TREX1 shows a binding affinity in the order of DNA/RNA hybrids > dsDNA >> dsRNA (*K*_d_ = 102.0 ± 8.0, 139.4 ± 17.2 and > 65 535 nM, respectively) (Figure 6B). This finding is consistent with the result presented earlier (Figure 1A) that the activity of mTREX1 in digesting DNA/RNA hybrids is slightly higher than that of degrading dsDNA. This result further corroborates that the natural substrates of TREX1 would involve DNA/RNA hybrids in addition to dsDNA. On the other hand, dsRNA is not a preferred substrate of TREX1, which can only digest the single-stranded 3′ overhang of dsRNA (Figure 1E). Notably, the *K*_d_ of ss19D1R annealed to complementary ssDNA is in between those of dsDNA and DNA/RNA hybrid, suggesting that the single 2′-OH does not cause steric hindrance to affect the binding of TREX1. In summary, our biochemical studies support the structural and protein dynamics analysis in this work, indicating that the 2′-OH affects the conformation and rigidity of RNA and thereby impacts mTREX1 binding. In general, the binding affinity of TREX1 for dsRNA is significantly lower than that for dsDNA, indicating that the conformational and mechanical differences mediated by 2′-OH are important properties for the distinct interactions of dsRNA and dsDNA with TREX1.

### RNase T shows a similar binding preference for the distinct catalytic activities over DNA and RNA substrates

To examine whether the aforementioned mechanism of TREX1 in distinguishing DNA and RNA substrates could be observed in other DEDDh exonucleases, RNase T was purified in a standard protocol (21), and the nuclease activity, binding ability and product-releasing rates were measured by using activity assay, electrophoretic mobility shift assay (EMSA) and ITF, respectively (Supplementary Figure S13). The results show that similar to TREX1, RNase T exhibits higher nuclease activity and binding affinity on the ssDNA substrate than those toward the ssRNA substrate. The *K*_d_ values of RNase T in binding with AMP and dAMP that do not have intra-chain hydrogen bonding, on the other hand, are similar, which is also consistent with the case of TREX1. This result suggests that intra-chain hydrogen bonding is also an important effector for the substrate binding of RNase T and potentially other exonucleases in the DEDDh family.

**Figure 7.**
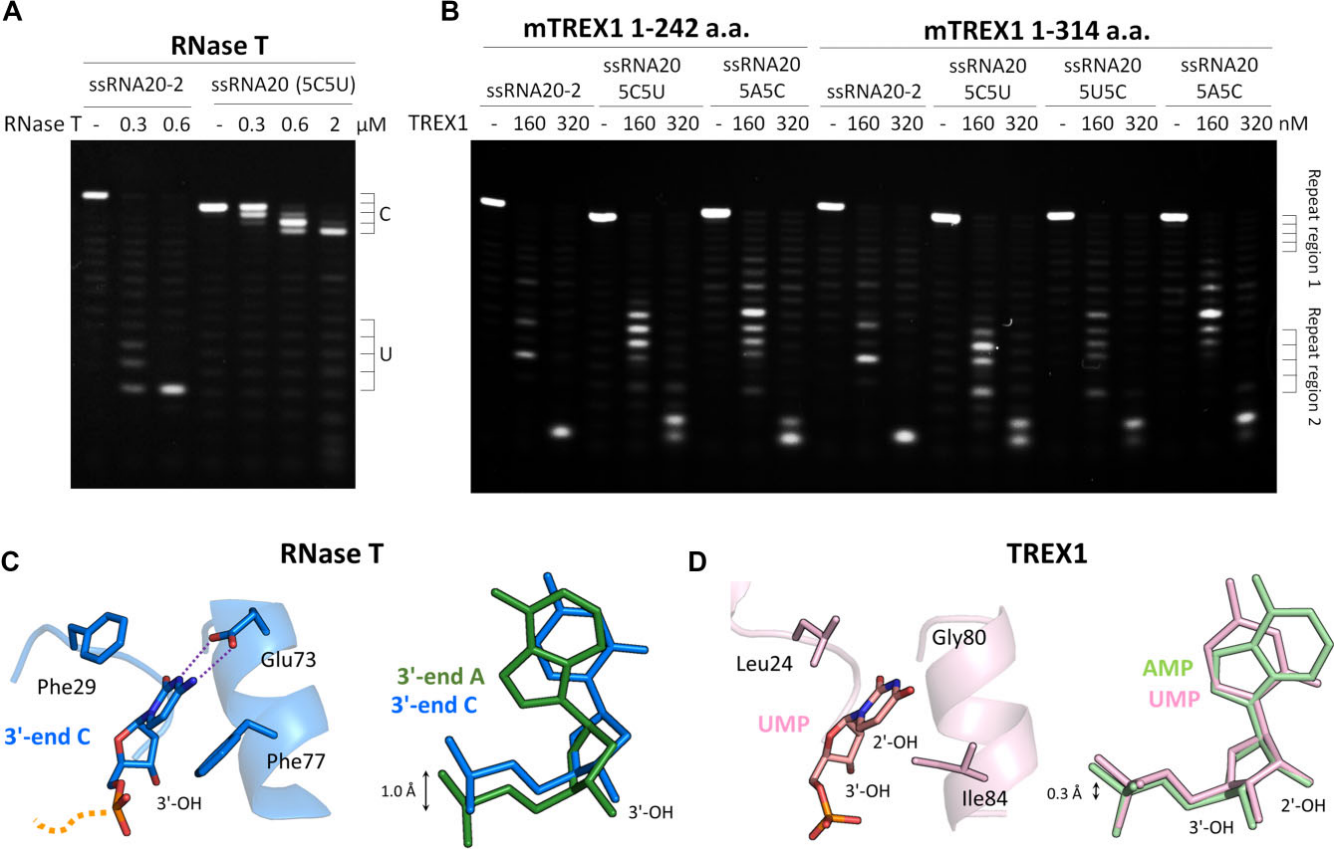
Biochemical and structural analysis of the sequence preference of TREX1. (**A**) Nuclease activity assay of RNase T on ssRNA with a random sequence (ssRNA 20-2) or poly(C)–poly(U) repetitive sequence (ssRNA 20 5C5U). (**B**) Nuclease activity assay of truncated and full-length mTREX1 on ssRNA with a random sequence (ssRNA 20–2) or repetitive sequences, such as ssRNA 20 5C5U, ssRNA 20 5A5C and ssRNA 20 5U5C. The cleavage patterns are similar. (**C**) The left panel shows the essential residues for the C-effect of RNase T. The right panel shows the structural comparison of RNase T binding with the preferred substrate (ssDNA with 3′ end A; PDB code:3V9X) versus binding with the non-preferred substrate (ssDNA with 3′ end C; PDB code:3NH0). As RNase T binds the non-preferred substrate, the scissile phosphate moves up by 1 Å and disrupts the catalytic environment. (**D**) The residues of TREX1 interacting with the 3′-ended nucleotide. Superposition of the nucleotides in the TREX1–AMP complex and TREX1–UMP complex structures. The positions of the scissile phosphates are almost identical.

TREX1 exhibited a sequence preference for processing RNA substrates, preferring adenine (A) over cytosine (C) and uracil (U), as indicated in previous studies (27). The *E. coli* homolog RNase T, on the other hand, showed a different behavior of solely excluding cytosine (C), and the specific structural features of RNase T can be used to understand the molecular mechanism (22,29). However, our biochemical and structural studies here illustrate a different pattern, unlike the previous work in which TREX1 showed a sequence preference for RNA substrates. To investigate this further, various ssRNA substrates with random sequences or five repeated A, C or U are synthesized. RNase T, an exonuclease with a C-effect that cannot digest RNA or DNA substrates containing repeated C (22,41,42), is used as a control. The results show that RNase T can digest the RNA substrates of random sequences but cannot process the RNA with five repeated C, indicating significant sequence preference (Figure 7A). Even at 2 μM RNase T, ∼3.3-fold the concentration that completely degrades the random-sequence RNA substrates, the poly(C) substrate remains intact. When the full-length and truncated mTREX1 are mixed with these RNA substrates, both mTREX1 proteins display non-sequence-dependent digestion patterns with no specific band accumulation at the repeated sequences. The digestion efficiency and cleavage band patterns of TREX1 are similar for all RNA substrates (Figure 7B). These findings suggest that TREX1 is an RNase without significant sequence preference.

Based on previous structural analysis, RNase T prevents the removal of the 3′-end C by two features (22,29), with Glu73 interacting with the base via two hydrogen bonds, and two C-filter phenylalanine residues (Phe29 and Phe73) stacking the 3′-end C, thereby causing the scissile phosphate of 3′-end C to move up and prevent the digestion by RNase T (Figure 7C). This mechanism indicates the three key residues and the movement of scissile phosphate of 3′-ended nucleotide as indicators for sequence specificity. However, the three residues are not conserved in TREX1, with Gly80 not interacting with the base region of the 3′-ended nucleotide, and Leu24 and Ile84 not serving as C-filter residues. According to another study (27), on the other hand, TREX1 could prefer the A base but not U. However, the structural analysis in this work shows that the positions of scissile phosphate of A and U are almost identical (Figure 7D). Both the biochemical and structural evidence collected here does not support that TREX1 has a significant sequence preference in processing RNA.

## Discussion

### The mechanism by which TREX1 distinguishes DNA and RNA substrates

Substrate binding affinity and product release rate are critical factors for the different DNase and RNase activities of TREX1. As a non-processive exonuclease, TREX1 distinguishes DNA and RNA substrates primarily with distinct binding strengths and exhibits similar product release rates. Structural analysis and critical affinity measurements show that the extra 2′-OH does not impair the release rate (Figures 2–4) for various products such as nucleosides and nucleotides. Therefore, substrate binding is likely to be the crucial factor for rendering the different DNase and RNase activities of TREX1.

According to the structural analysis conducted in this work, the 2′-OH at the 3′-end nucleotide of a substrate interacts with TREX1 through several hydrogen bonds and does not lead to steric hindrance (Figure 2). Furthermore, TREX1 can also accommodate the other 2′-OH groups besides the last nucleotide of ssRNA substrates without causing steric hindrance (27), indicating that steric hindrance is not an important factor. Our all-atom MD simulations and binding assays using single-stranded DNA–RNA junctions as substrates (Figure 6B) indicate that the 2′-OH in RNA mediates higher conformational rigidity in the polymer chain and leads to weaker binding between TREX1 and RNA substrates. Additional studies are conducted here to show that this mechanism is also important in RNase T and potentially other exonucleases.

For the clear inability of TREX1 to process dsRNA, the A-form structure of dsRNA (43) involves significantly different rigidities compared with those in the B-form structure of dsDNA (40). Recent studies also highlight that the RNA secondary structure affects the binding with protein molecules (44). Furthermore, when TREX1 binds to dsDNA, the disease-related Arg128 is inserted into its minor groove and interacts with the non-scissile strand via several hydrogen bonds (Supplementary Figure S14) (3). Formation of this interaction between TREX1 and dsRNA could thus be affected due to the different structure and mechanical properties, leading to low binding affinity and nuclease activity. Interestingly, the activity levels of DNA/RNA hybrids are similar to those of dsDNA, which calls for further development of mechanistic understanding.

### The cellular function related to the RNase activity of TREX1

While ssDNA and dsDNA are both TREX1 substrates, the double-stranded structure of dsRNA blocks the RNase activity even when the TREX1 concentration is raised to 25-fold the necessary concentration for digesting ssRNA or when HMGB-2 is added to enhance the activity for digesting double-stranded substrates (Figure 1F). Similar to the case of RNase T (22,29), the property of TREX1 in processing the single-stranded 3′ overhang but stopping at the double-stranded structure would be an important feature in RNA maturation, but the detailed participation of TREX1 requires further studies. On the other hand, even though both TREX1 and RNase T cannot digest dsRNA, the molecular mechanisms are different. Firstly, unlike RNase T, TREX1 does not show sequence specificity toward RNA substrates (Figure 7). Secondly, RNase T is a dimeric DEDDh exonuclease, and the two monomers bind the scissile strand and the non-scissile strand of a double-stranded substrate for digestion (21,22,29). TREX1 is also a dimeric DEDDh exonuclease, but the arrangement of the two monomers adopts a different orientation (28) such that the scissile or non-scissile strand of different substrates is bound independently. The structural origin of dsRNA blocking the activity of TREX1 and RNase T is thus expected to be different, which calls for additional TREX1–RNA complex structures to reveal the details.

### TREX1 is involved in the DNA/RNA hybrid metabolism

TREX1 mutations were shown to lead to AGS disease and the accumulation of DNA/RNA hybrids in cells (30). On the other hand, HIV utilizes TREX1 to degrade non-productive HIV reverse transcripts to evade the immune system (9). Interestingly, when the HIV-1 DNA/RNA hybrid is imported into Trex1^−/−^ MEFs, the viral-induced immune activation is turned on, suggesting that the HIV DNA/RNA hybrids are also non-productive HIV reverse transcripts and that TREX1 can play the role of processing the unwanted genetic material (9). In addition, the nuclease activity of TREX1 is related to controlling retroelement transposition (4,13). These studies show the possible cellular pathways of TREX1 in processing DNA/RNA hybrids.

Another interesting question is whether TREX1 and another AGS-related nuclease, RNase H2, work together in processing DNA/RNA hybrids. Mutations in TREX1 and RNase H2 were shown to lead to very similar autoimmune diseases, suggesting that they function in the same pathway and share common DNA/RNA hybrid substrates derived from endogenous retroelements (45). The exonuclease activity of TREX1 combining the endonuclease activity of RNase H2 may increase the efficiency in processing DNA/RNA hybrids. A potential mode of cooperation is that TREX1 processes the DNA and RNA strands of the DNA/RNA hybrid after RNase H (4,46) to increase efficiency. Consistent with this hypothesis, our nuclease activity measurements show that the activity levels of the two nucleases are similar (Figure 1; Supplementary Figure S2). However, TREX1 is located at the ER in the cytoplasm, but RNase H2 is a nuclear protein. As such, these two nucleases might work sequentially, with the DNA/RNA hybrid exported to the cytoplasm for TREX1 digestion after the endonucleolytic process of RNase H2. Another possibility is that TREX1 translocates into the nucleus to work with RNase H2, as the translocation property of TREX1 was observed in the presence of genotoxic stresses (12–14). On the other hand, RNase H2 was shown to recognize and digest the DNA/RNA hybrid in the R-loop to prevent DNA breaks and inflammation and maintain genomic integrity (47,48). Recent studies show that the R-loop, a type of DNA/RNA hybrid intermediate, is also associated with DNA repair, especially in the repair of double-strand breaks (DSBs) (49–52). Similar to TREX1, these studies show that RNase H2 may also be associated with various DNA repair pathways. Interestingly, SAMHD1, the third protein related to DNA/RNA hybrid accumulation and AGS, acts at stalled replication forks and is important for replication restart under replication stress response (53). Whether these three AGS-related nucleic acid-binding proteins working in similar DNA repair pathways, and how the DNA repair pathways interfere with the interferon induction-related immune response are practical issues that need to be addressed further.

## Supplementary Material

gkad910_Supplemental_File

## Data Availability

The datasets generated and analyzed in the current study are available from the corresponding author upon reasonable request. The coordinates of all the structures addressed in this work are available in the Protein Data Bank (wwPDB), including the structures determined in this study, under accession code 8HCC (mTREX1–RNA product complex), 8HCD (mTREX1–DNA product complex), 8HCE (mTREX1–CMP complex), 8HCF (mTREX1–UMP complex), 8HCG (mTREX1–dAMP complex) and 8HCH (mTREX1–uridine complex), and in previous studies (PDB IDs 2OA8, 2IOC, 2O4G, 3NH0, 3V9X, 5YWT, 5YWU, 5YWS and 6A45).
