# Supplementary material for: Molecular insight into the specific enzymatic properties of TREX1 revealing the diverse functions in processing RNA and DNA/RNA hybrids
Source: Nucleic Acids Res. 2023 Oct 23;51(21):11927–40. doi: 10.1093/nar/gkad910 (PMC10681709; doi:10.1093/nar/gkad910)
Supplement: gkad910_Supplemental_File [file gkad910_supplemental_file.pdf]

Supplementary information

For

Molecular Insight into the Specific Enzymatic Properties of TREX1 Revealing the Diverse Functions in Processing RNA and DNA/RNA Hybrids

Kuan-Wei Huang[1,2,†], Chia-Yun Wu[1,2,†], Shu-Ing Toh[1,2], Tung-Chang Liu[1,2], Chun-I Tu[1,2], Yin-Hsin Lin[1], An-Ju Cheng[1], Ya-Ting Kao[1,2,3,4], Jhih-Wei Chu[1,2,3,4 *] and Yu-Yuan Hsiao[1,2,3,4,5,6 *]

[1] Department of Biological Science and Technology, National Yang Ming Chiao Tung University, Hsinchu, 30068, Taiwan.

[2] Institute of Molecular Medicine and Bioengineering, National Yang Ming Chiao Tung University, Hsinchu, 30068, Taiwan

[3] Institute of Bioinformatics and Systems Biology, National Yang Ming Chiao Tung University, Hsinchu, 30068, Taiwan.

[4] Center for Intelligent Drug Systems and Smart Bio-devices (IDS^{2}B), National Yang Ming Chiao Tung University, Hsinchu, Taiwan

[5] Drug Development and Value Creation Research Center, Center for Cancer Research, Kaohsiung Medical University, Kaohsiung, Taiwan

[6] Department of Biomedical Science and Environmental Biology, Kaohsiung, 807378, Taiwan

† Joint Authors； K-W. H. and C-Y. W. contributed equally.

* To whom correspondence should be addressed. Jhih-Wei Chu：Tel: +886-3-571-2121 ext.56996; Fax: +886-3-5729288; Email: jwchu@nycu.edu.tw ; Yu-Yuan Hsiao：Tel: +886-3-571-2121 ext.56999; Fax: +886-3-5729288; Email: mike0617@nycu.edu.tw

Supplementary Methods
Supplementary Tables： 1-2
Supplementary Figures： 1-14

Supplementary Methods

Expression and purification of mHMGB2

The wild-type mouse HMGB2 (mHMGB2, 1-210 a.a) gene was cloned into a pET28a vector and expressed in *E. coli* BL21-CodonPlus (DE3)-RIPL strain. *E. coli* cells were cultured in Luria Broth (LB) medium at 37 °C supplemented with 35 μg/ml chloramphenicol, 25 μg/ml streptomycin, and 50 μg/ml kanamycin to OD_{600} of 0.5-0.6 and then induced by 1 mM isopropyl β-D-1-thiogalactopyranoside at 18 °C for 20 h. The cells were collected through centrifugation at 8,000 rpm for 30 min at 4 °C and further lysed through sonication in 50 mM Tris-HCl pH 8.0, 300 mM NaCl. The cell debris was clarified through centrifugation at 13,000 rpm at 4 °C for 30 min, and the supernatant was loaded into an affinity column (HiTrap TALON crude 5 ml, GE Healthcare) and purified by standard protocol. Target proteins were further purified by an anion-exchange column (HiTrap Heparin HP 5 ml, GE Healthcare) and a size-exclusion column (HiLoad™ 16/60 Superdex™ 75 prep grade, GE Healthcare). Purified mHMGB2 was concentrated to at least 10mg/mL in 50 mM Tris-HCl pH 7.0, 300 mM NaCl, and stored at -20 °C until use.

Expression and purification of mRNaseH1 and mRNaseH2

The truncated mouse RNaseH1 (mRNaseH1, 27-285 a.a) gene was cloned into a pET22b vector and expressed in *E. coli* B834 (DE3) pLysS strain. *E. coli* cells were cultured in Luria Broth (LB) medium at 37 °C supplemented with 35 mg/ml chloramphenicol and 100 mg/ml ampicillin to OD_{600} of 0.4-0.6 and then induced by 1 mM isopropyl β-D-1-thiogalactopyranoside at 18 °C for 18 h. The cells were collected through centrifugation at 6,000 rpm for 30 min at 4 °C, and further lysed through sonication in 50 mM Tris-HCl pH 8.0, 300 mM NaCl. The cell debris was clarified through centrifugation at 13,000 rpm at 4 °C for 30 min and the supernatant was loaded into an affinity column (HisTrapFF 5 ml, GE Healthcare) and purified by standard protocol. Target proteins were further purified by an ion-exchange column (HiTrap SP FF 5 ml, GE Healthcare) and a size-exclusion column (HiLoad™ 16/60 Superdex™ 75 prep grade, GE Healthcare). Purified truncated mRNaseH1 was concentrated to at least 7 mg/mL in 50 mM CH_3COONa pH 4.5, 500 mM NaCl, and stored at -20 °C until use.

The wild-type mouse RNaseH2A and RNaseH2BC genes were cloned into a pETDuet vector and pRSFDuet vector, respectively, and co-expression in *E. coli* B834(DE3) pLysS strain. *E. coli* cells were cultured in LB medium at 37 °C supplemented with 50 mg/ml kanamycin, 35 mg/ml chloramphenicol, and 100 mg/ml ampicillin to an OD_{600} of 0.6-0.7 and then induced by 1 mM isopropyl β-D-1-

thiogalactopyranoside at 18 °C for 18 h. The cells were harvested through centrifugation and further lysed through sonication in 50 mM Tris-HCl, 300 mM NaCl, pH 8.0. The lysate was clarified through centrifugation at 13 000 rpm at 4°C for 20 min. The supernatant was loaded into an affinity column (HiTrap™ TALON crude 5 ml, GE Healthcare) and purified by standard protocol. Target proteins were further purified by an ion-exchange column (HiTrap™ HP 5 ml, GE Healthcare), an ion-exchange column (HiTrap™ SP HP 5 ml, GE Healthcare), and a size-exclusion column (HiLoad™ 16/60 Superdex 200 prep grade, GE Healthcare). Purified wild-type mRNaseH2ABC complex was concentrated to at least 7.5 mg/mL in 50 mM Tris-HCl pH 7.0, 300 mM NaCl, and stored at -20 °C until use.

<u>Electrophoretic Mobility Shift Assay</u>

Gel shift assays were conducted to determine the DNA and RNA binding affinities of RNase T. After incubating RNase T in a solution containing 120 mM NaCl, 50 mM EDTA, and 20 mM Tris-HCl, pH 7.0 for 10 minutes on ice, 5’-FAM-labeled ssDNA and ssRNA substrates (0.5 μM) were added and further incubated for an additional 10 minutes on ice. The concentrations of RNase T used were 2.5, 5, 10, 20, and 40 μM. The resulting samples were separated using 10% TBE native PAGE and visualized with a fluorescence detector.

Supplementary Table 1. Substrates for biochemical studies

Substrate	Sequence
ssDNA20 (20 nt)	5'- FAM - **ACTGGACAAATACTCCGAGG** -3'
ssRNA20 (20 nt)	5'- FAM - ACUGGACAAAUACUCCGAGG -3'
dsDNA20 (20 bp)	5'- FAM - **ACTGGACAAATACTCCGAGG** -3'<br>3' - **TGACCTGTTTATGAGGCTCC** -5'
dsRNA20 (20 bp)	5'- FAM - ACUGGACAAAUACUCCGAGG -3'<br>3' - UGACCUGUUUAUGAGGCUCC -5'
**DNA***/RNA Hybrid (20 bp)	5'- FAM - **ACTGGACAAATACTCCGAGG** -3' **DNA**<br>3' - UGACCUGUUUAUGAGGCUCC -5' RNA
**DNA**/RNA* Hybrid (20 bp)	5'- FAM - ACUGGACAAAUACUCCGAGG -3' RNA<br>3' - **TGACCTGTTTATGAGGCTCC** -5' **DNA**
RNA-**DNA** junction (20 nt)	5'- FAM - ACUGGACAAA**TACTCCGAGG** -3'
Y-structural dsRNA20	5'- FAM - ACUGGACAAAUACUCCGAGGUUGGG -3'<br>3' - UGACCUGUUUAUGAGGCUCCUUAAA -5'
dsRNA20 (3′-overhang)	5'- FAM - ACUGGACAAAUACUCCGAGGUUGGG -3'<br>3' - UGACCUGUUUAUGAGGCUCC -5'
dsRNA20 (5′-overhang)	5'- FAM - ACUGGACAAAUACUCCGAGG -3'<br>3' - UGACCUGUUUAUGAGGCUCCUUAAA -5'
ss**DNA**19+RNA1(ss19D1R)	5'- FAM - **ACTGGACAAATACTCCGAG** G -3'
ds**DNA**19+RNA1<br>(ss19D1R/ssDNA20)	5'- FAM - **ACTGGACAAATACTCCGAG** G -3'<br>3' - **TGACCTGTTTATGAGGCTC C** -5'
ss**DNA**17+RNA3(ss17D3R)	5'- FAM - **ACTGGACAAATACTCCG** AGG -3'
ssRNA20-2	5'- FAM - CAAACUGUGAUGUGAUGAAC -3'
ssRNA20 5C5U	5'- FAM - GCAUAUUUUUACUCGCCCCC -3'
ssRNA20 5A5C	5'- FAM - GCAUACCCCCACUCGAAAAA -3'
ssRNA20 5U5C	5'- FAM - GCAUACCCCCACUCGUUUUU -3'

1. **boldface :** DNA substrate
2. bottom line : unpaired region

Supplementary Table 2. Crystallization conditions of mTREX1-nucleotide/nucleoside complexes

**mTREX1-RNA product complex (AMP)**	
Protein：His-tagged truncated mTREX1 (1-242 a.a.)<br>Input RNA：5′- AACC -3′<br>Time of growth：3 weeks<br>Condition ： 0.2 M Magnesium acetate tetrahydrate, 0.1 M Sodium cacodylate trihydrate pH 6.5, 20 % w/v Polyethylene glycol 8,000	Nucleotide in the structure：AMP
**mTREX1-DNA product complex (dCMP and dGMP)**	
Protein：His-tagged truncated mTREX1 (1-242 a.a.)<br>Input DNA：5′ - GAGGCCCTCTTTAGGGCCAG - 3′<br>Time of growth：3 weeks<br>Condition：0.2 M Ammonium sulfate, 0.1 M Sodium cacodylate trihydrate pH 6.5, 30 % w/v Polyethylene glycol 8,000	Nucleotide in the structure：<br>dCMP and dGMP
**mTREX1-CMP complex**	
Protein：His-tagged truncated mTREX1 (1-242 a.a.)<br>Input nucleotide：Cytidine 5'-Monophosphate<br>Time of growth：3 days<br>Condition：0.2 M Ammonium sulfate, 0.1 M HEPES pH 7.5, 25% w/v Polyethylene glycol 3,350	Nucleotide in the structure：CMP
**mTREX1-UMP complex**	
Protein：His-tagged truncated mTREX1 (1-242 a.a.)<br>Input nucleotide：Uridine 5'-monophosphate<br>Time of growth：6 weeks<br>Condition：0.2 M Ammonium sulfate, 0.1 M HEPES pH 7.5, 25 % w/v Polyethylene glycol 3,350	Nucleotide in the structure：UMP
**mTREX1-dAMP complex**	
Protein：His-tagged truncated mTREX1 (11-242 a.a.)<br>Input DNA/RNA hybrid：<br>DNA：5'- TCCCTTTATCTCTCTTA -3'[a]<br>RNA：3'- ACAGGGAAAUAGAGAGA -5'<br>Time of growth：2 weeks<br>Condition：0.1 M HEPES pH 7.5, 40 % v/v Polyethylene glycol 400	Nucleotide in the structure：dAMP
**mTREX1-Uridine complex**	
Protein：His-tagged truncated mTREX1 (11-242 a.a.)<br>Time of growth：2 weeks<br>Condition：12.5 % v/v MPD; 12.5 % PEG 1000; 12.5 % w/v PEG 3350, 0.2 % w/v Cytidine, 0.2 % w/v Inosine, 0.2 % w/v Ribavirin, 0.2 % w/v Thymidine, 0.2 % w/v Uridine, 0.1M Imidazole, 0.1M MES monohydrate (acid) pH 6.5	Nucleoside in the structure：Uridine

[a] The underline regions are paired regions of DNA/RNA hybrid substrates

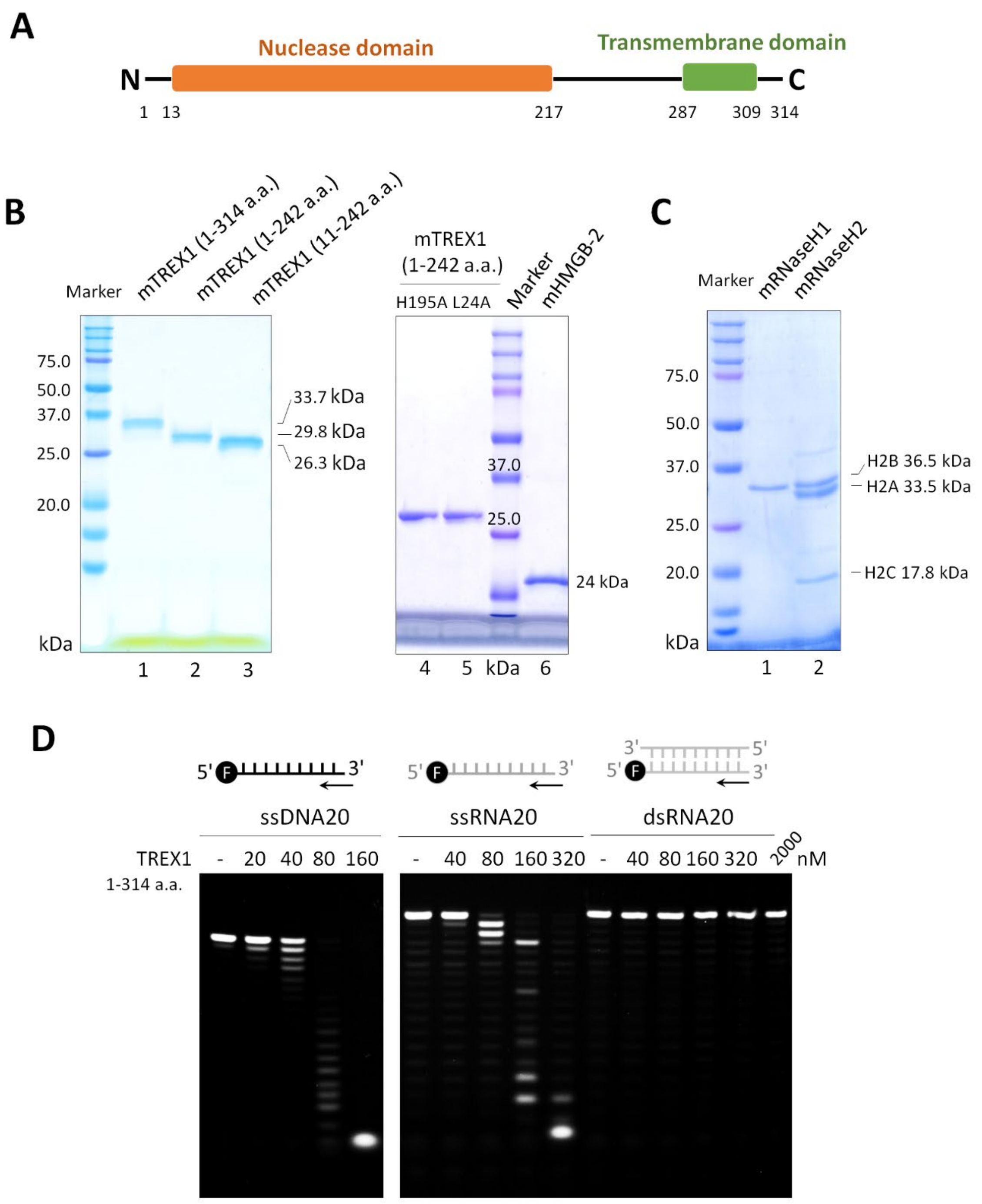

Supplementary Figure 1. The purification and activity assays of mTREX1, mRNase H1, and mRNase H2. (A) The domain structure of mTREX1. (B)(C) The recombinant full-length, truncated, and mutated mTREX1, mHMGB-2, mRNase H1, and mRNase H2 were purified and protein purity was analyzed by SDS-PAGE. (D) Nuclease activity assays of full-length mTREX1 on digesting ssDNA (20 mer), ssRNA (20 mer), and dsRNA (20 bp). The activity property is similar to truncated mTREX1.

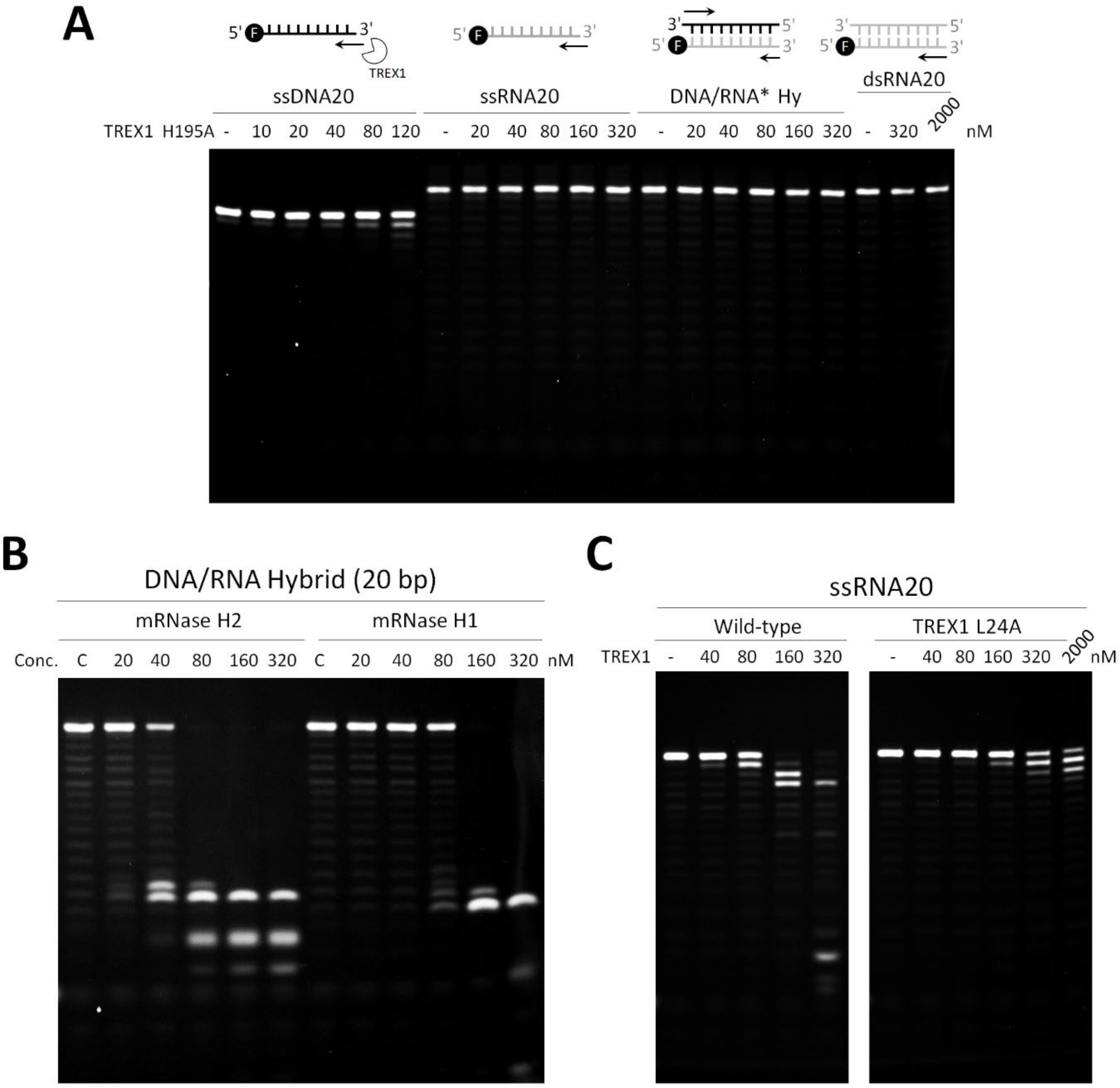

Supplementary Figure 2. The nuclease activity assays of mTREX1 mutants, mRNase H2 and mRNase H1. (A) (C) The nuclease activity assays of truncated mTREX1 mutants (1-242 a.a.), such as H195A and L24A. (B) The nuclease activity assays of mRNase H2 and mRNase H1. mRNase H2 or mRNase H1 mixed with DNA/RNA hybrid substrate (20 bp) in 2 mM $MgCl_2$, 20 mM Tris-HCl pH 7.0, 120 mM NaCl in 37°C for 30min. The result was analyzed by the standard protocol shown in Materials and Methods.

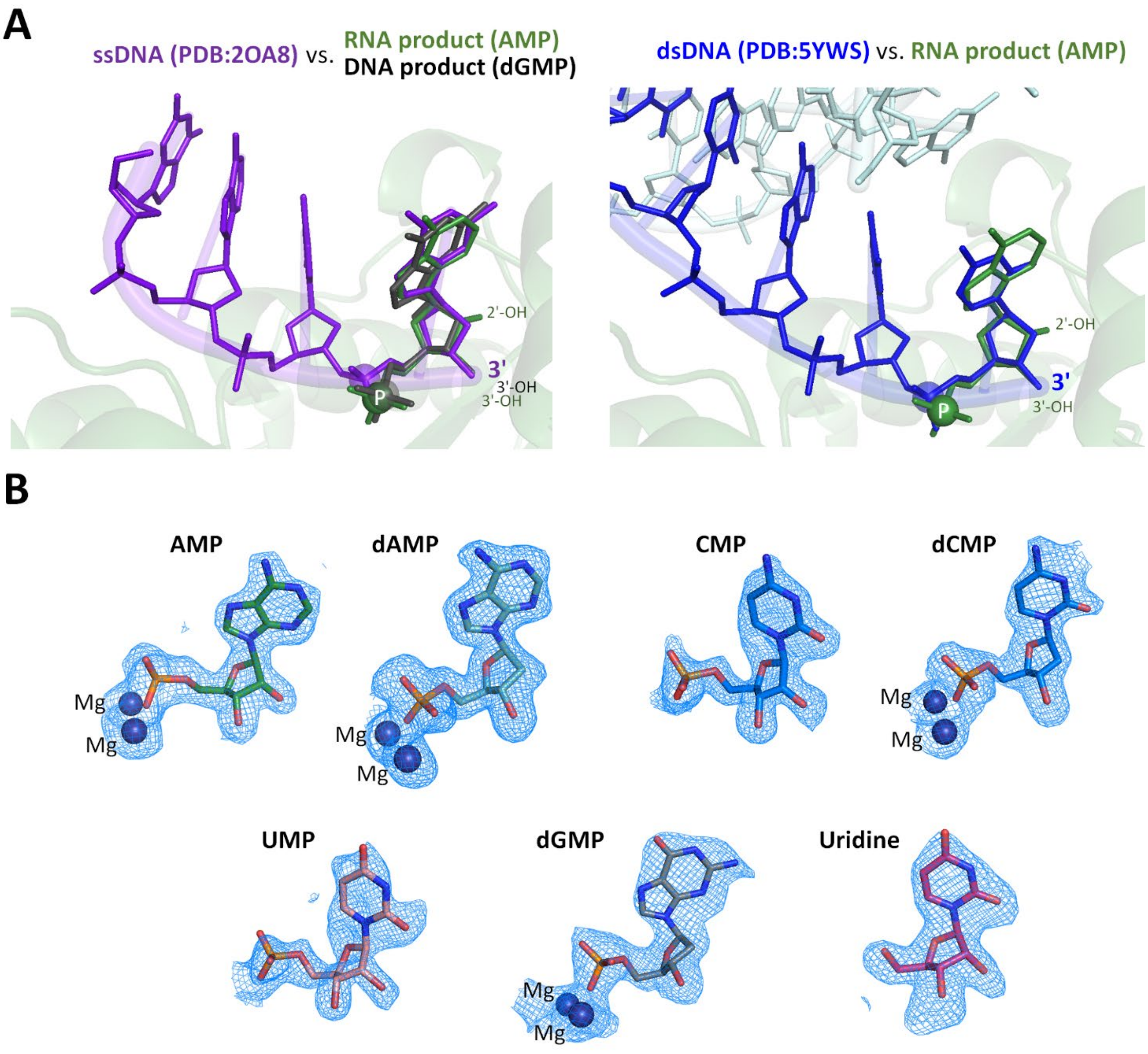

Supplementary Figure 3. Superposition of TREX1-RNA product complex and TREX1-DNA product complex with TREX1-substrate complexes, and the omitted electron density maps of nucleotides and nucleosides. (A) The structural alignment of TREX1-RNA product complex (AMP; colored in green) and TREX1-DNA complex (dGMP; colored in black) with TREX1-ssDNA (left panel; PDB code: 2OA8) or TREX1-dsDNA complexes (right panel; PDB code: 5YWS). The scissile phosphates are shown as ball-like structures. (B)The omitted electron density map (Fo − Fc, 2.0 σ) of nucleotides and nucleosides in the mTREX1-RNA product complex (AMP), mTREX1-DNA product complex (dCMP and dGMP), mTREX1-nucleotide complex (UMP, CMP, and GMP), and mTREX1-uridine complex structures. The blue balls are Mg ions.

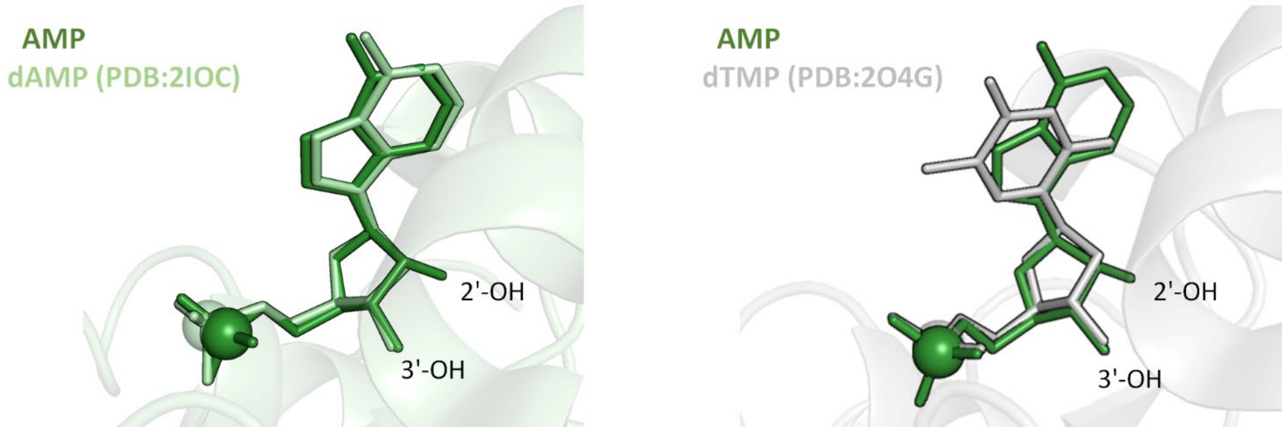

Supplementary Figure 4. The superposition of the RNA product (AMP) in this study and the deoxyribonucleotides (dAMP and dTMP) from previous studies. The Left and right panels show the results of the structural comparison of the RNA product (AMP) in the TREX1-RNA product complex with the deoxynucleotides in the TREX1-dAMP and TREX1-dTMP complex, respectively. The phosphates of nucleotides are displayed as balls.

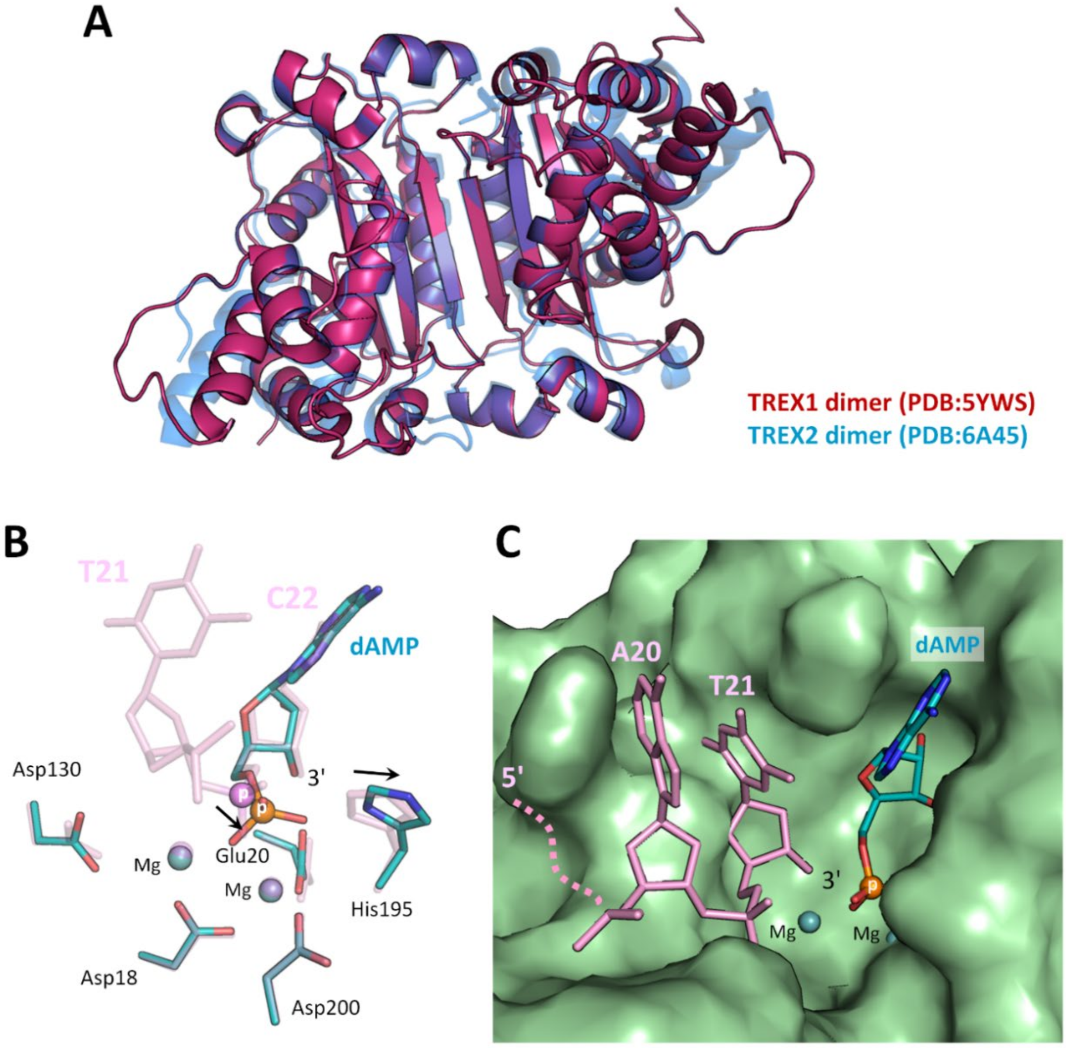

Supplementary Figure 5. Active site structure of mTREX1-DNA product complex. (A) The structure alignment of TREX1 (PDB code: 5YWS) and TREX2 (PDB code: 6A45). (B)The superposition of the two active sites in mTREX1-substrate complex (mTREX1-Y structured DNA complex; PDB code: 5YWS) and mTREX1-DNA product complex (dAMP). The substrate complex is colored pink, and the T21 and C22 mean thymine 21, and cytosine 22 are the last two nucleotides (21st and 22nd) in the 3'-end. The scissile phosphates are displayed as pink or orange balls and labeled “P”. The two Mg^{2+} are displayed as pink and light blue balls, respectively. (C) The structural model of the transition state is between substrate cleaving and product release. We used the product dAMP to replace C22 to mimic the intermediate state that TREX1 broke the phosphodiester bond at the 3'-ended nucleotide of the substrate. mTREX1 protein is displayed as surface mode. The cleaved product, dAMP, is deeply in the product pocket. The dAMP cannot be released before the remaining substrate (pink) is released. Therefore, TREX1 is a non-processive nuclease and can not process substrates continuously.

A

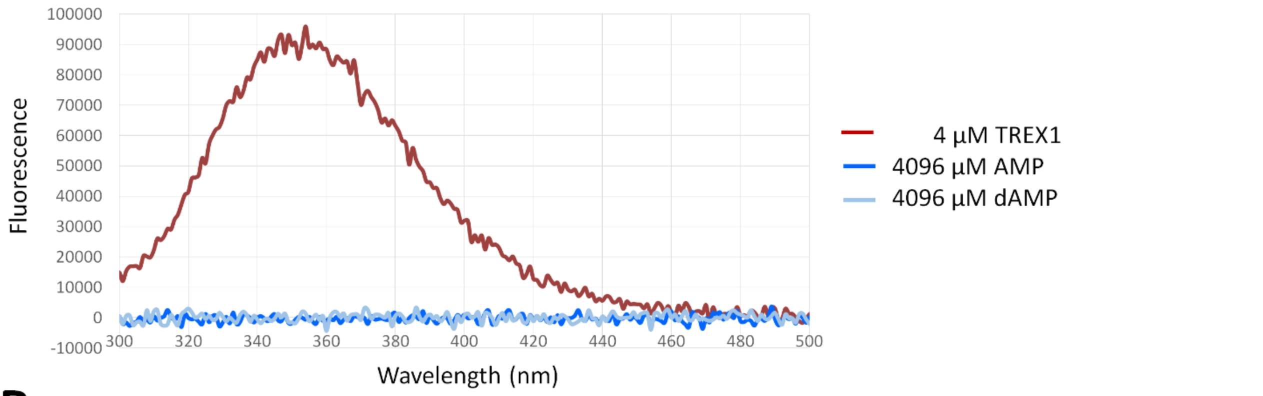

B

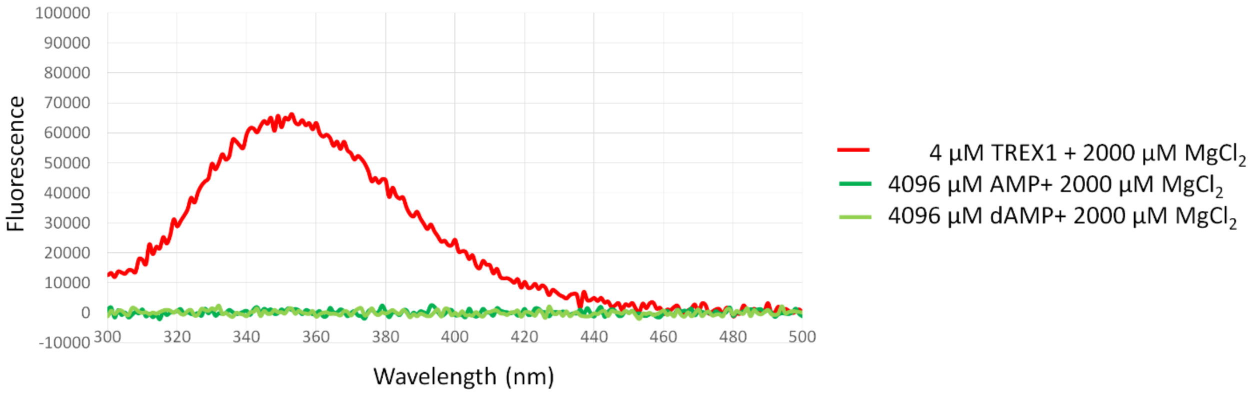

Supplementary Figure 6. The control experiment of Intrinsic Tryptophan Fluorescence (ITF) measurement of the K_d. The ITF signal of 4 µM mTREX1 or nucleotides in 4096 µM, such as AMP and dAMP, in the presence or absence of 2 mM $MgCl_2$ was examined. Upon excitation at 270 nm, no significant fluorescence emissions between 290 and 500 nm were observed from the aromatic ring of nucleotides.

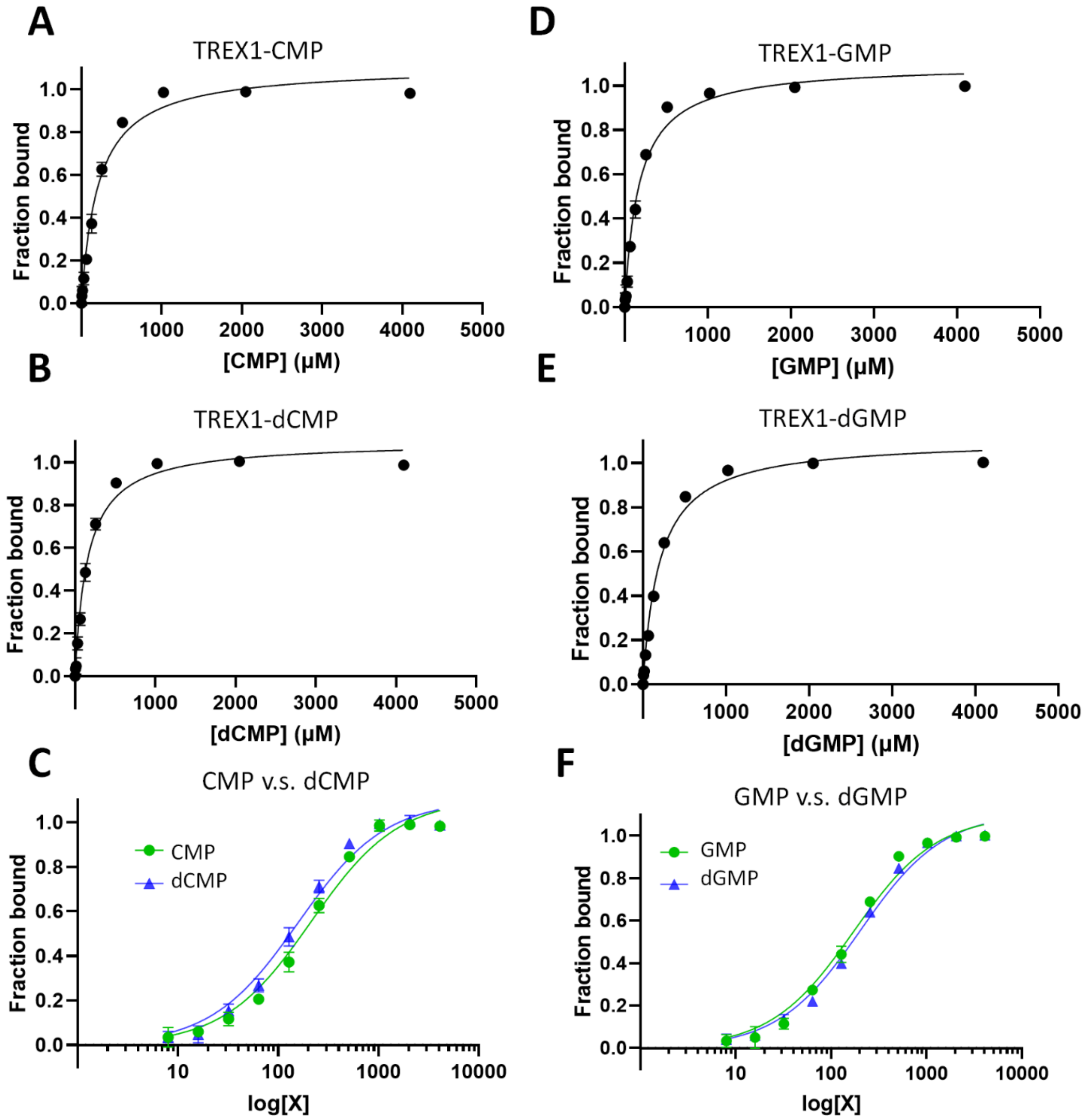

Supplementary Figure 7. The Intrinsic Tryptophan Fluorescence (ITF) measurement of the K_d between mTREX1 and various nucleotides in the presence of 2 mM $MgCl_2$. (A) (B) (D) (E) Quantification of the affinity (K_d) between mTREX1 and various ribonucleotides and deoxyribonucleotides, such as CMP, dCMP, GMP, and dGMP. Dissociation constants (K_d) were calculated using a one-site binding mode. (C) (F) The comparison of the binding between CMP and dCMP, or GMP, and dGMP. X in the log [X] is the concentration of CMP, dCMP, GMP, or dGMP.

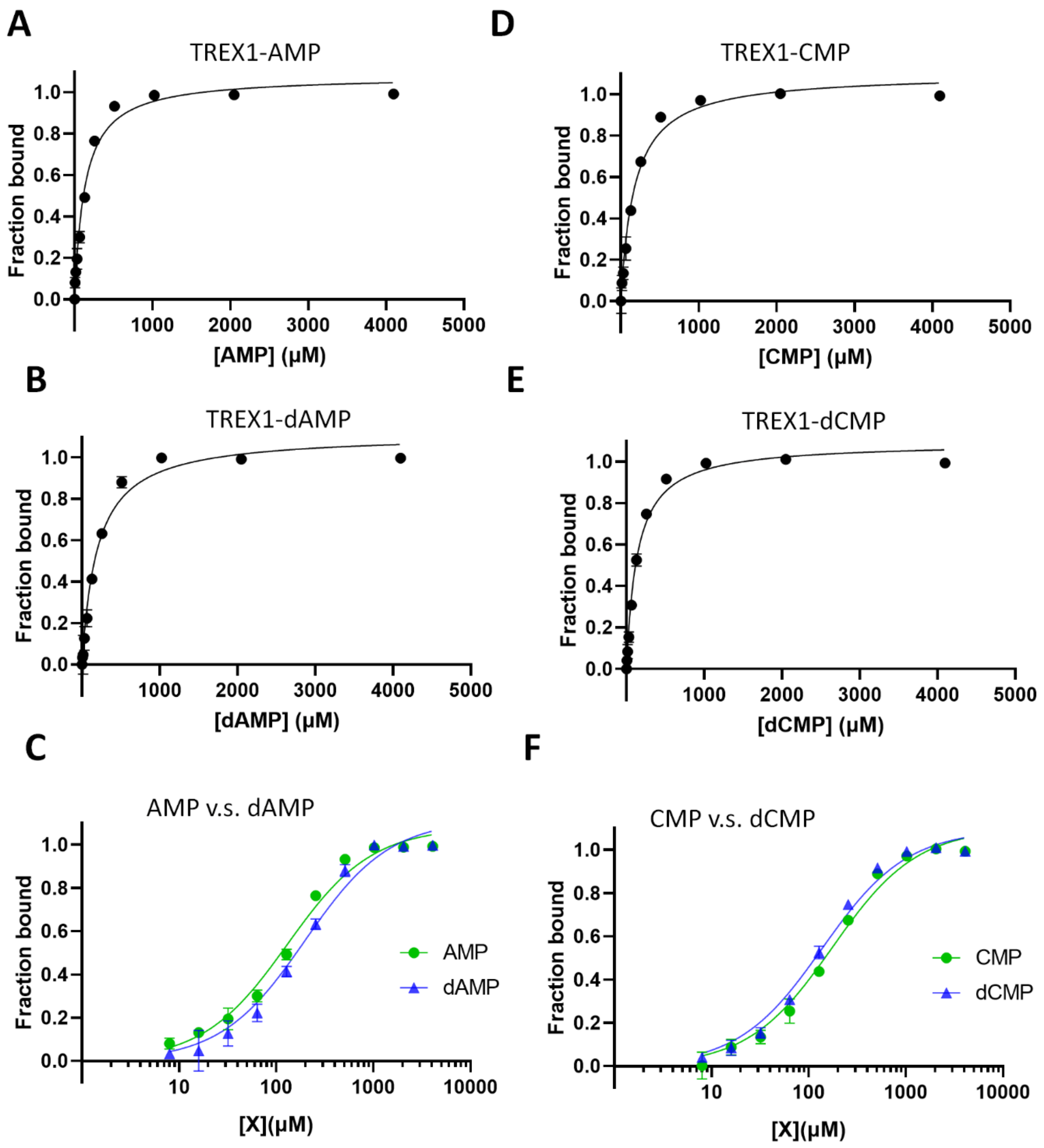

Supplementary Figure 8. The Intrinsic Tryptophan Fluorescence (ITF) measurement of the K_d between mTREX1 and various nucleotides in the absence of $MgCl_2$. (A) (B) (D) (E) Quantification of the affinity (K_d) between mTREX1 and various ribonucleotides and deoxyribonucleotides, such as AMP, dAMP, CMP and dCMP in the absence of $MgCl_2$. Dissociation constants (K_d) were calculated using a one-site binding mode. (C) (F) The comparison of the binding between AMP and dAMP, or CMP, and dCMP. X in the log [X] is the concentration of AMP, dAMP, CMP, or dCMP.

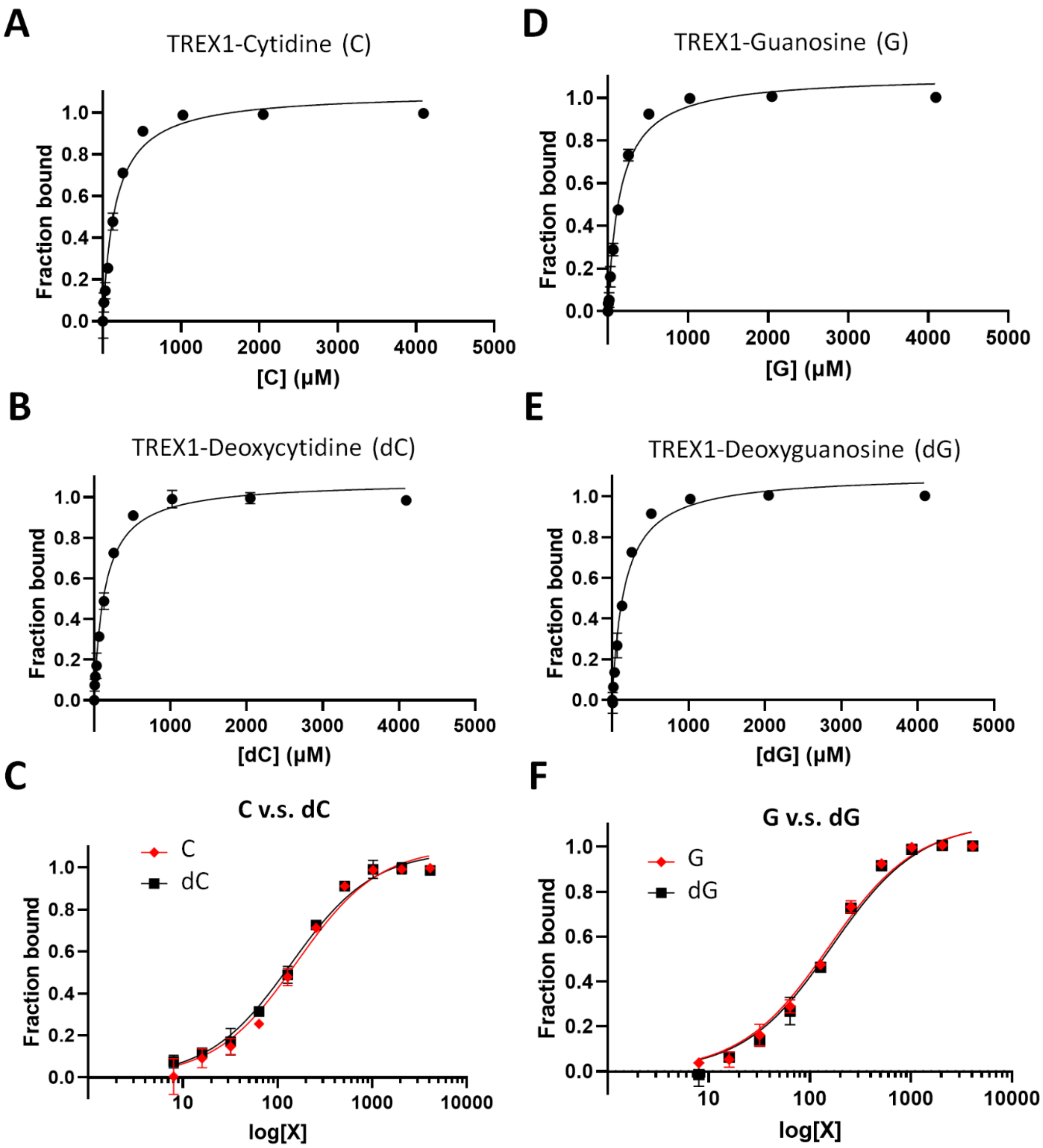

Supplementary Figure 9. The ITF measurement of the K_d between mTREX1 and various nucleosides in the presence of 2 mM $MgCl_2$. (A) (B) (D) (E) Quantification of the affinity (K_d) between mTREX1 and nucleosides, including cytidine (C), deoxycytidine (dC), guanosine (G), and deoxyguanosine (dG). Dissociation constants (K_d) were calculated using a one-site binding model. (C) (F) The comparison of the binding between C and dC or G and dG. X in the log [X] is the C, dC, G, or dG concentration.

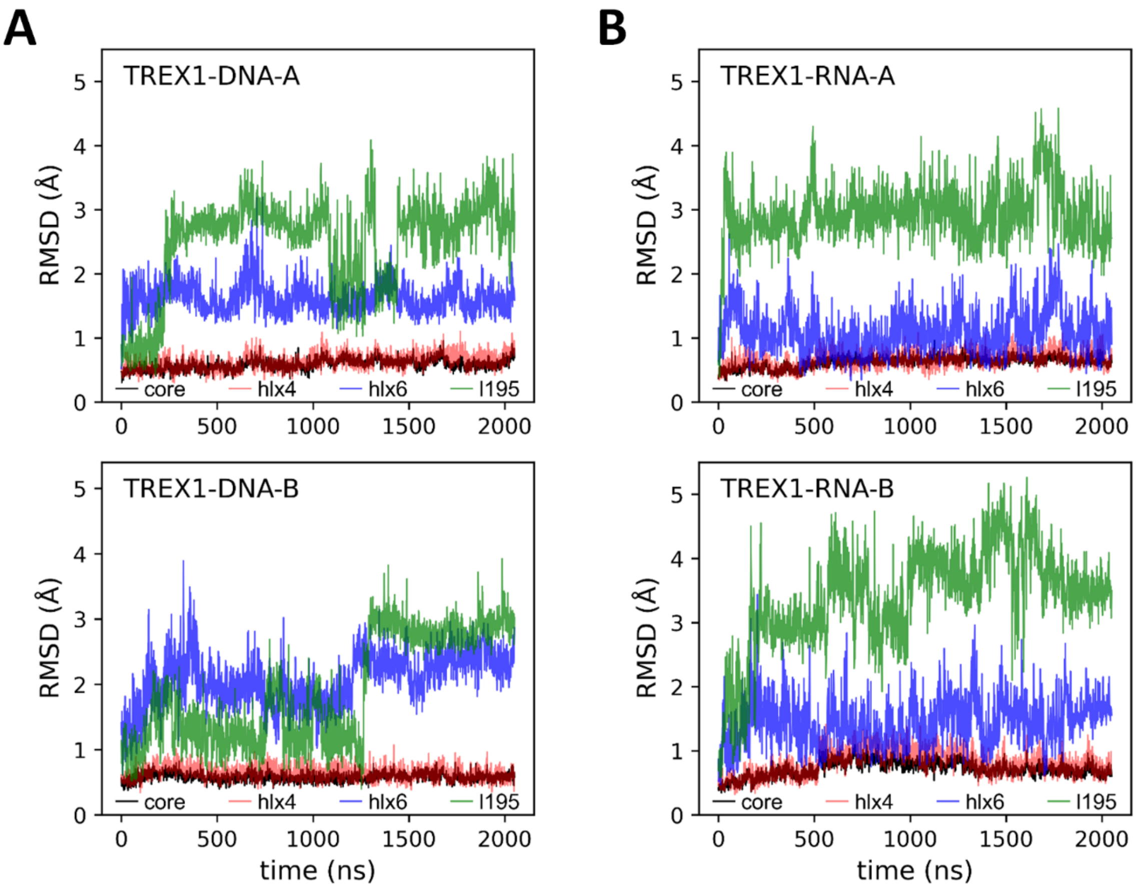

Supplementary Figure 10. The C_α RMSD in Å of TREX1 structural segments that exhibit contacts with the DNA or RNA substrate in the 2 μs production run of the all-atom MD simulation. The reference structure of TREX1 is 5YWS. The core contains residues 14-21, 34-40, 65-70, 130-140, and 198-210; the former three are β-strands and the latter two are helices. Asp18, Glu20, Asp130, Asp200, and His195 are the DEDDh residues. The hlx4 segment contains residues 124-140 and involves a helix in the core. The hlx6 segment contains residues 174-188 and has a helix around the core. The l195 segment is a loop of residues 189-197 where the general base His195 situates. The RMSD versus simulation time profile is black for core, red for hlx4, blue for hlx6, and green for l195. (a) The profiles of chain A and chain B in the TREX1-DNA simulation. (b) The profiles of chain A and chain B in the TREX1-RNA simulation

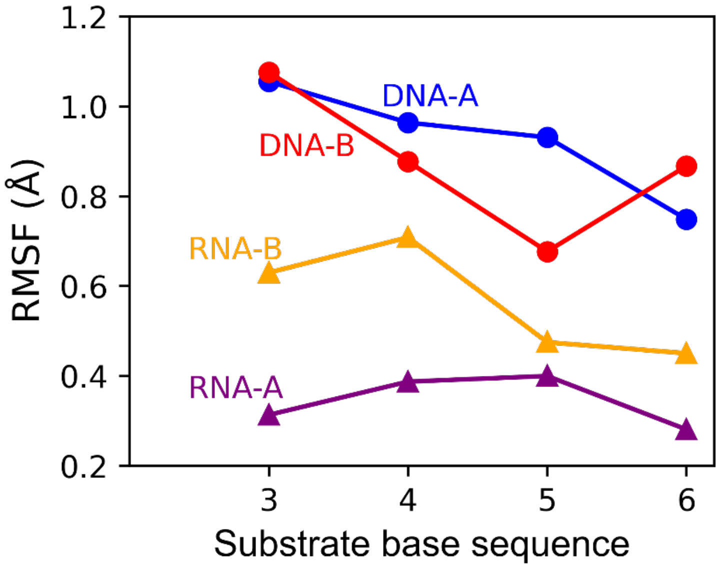

Supplementary Figure 11. RMSF in Å of the ssDNA or ssRNA chain in the all-atom MD simulation with TREX1. Trajectory snapshots in the 2 μs production run of each simulation are used for the calculation. The RMSF is calculated for each base according to the position of the backbone phosphorus atom. The RMSF-versus-base profile is calculated for the segment of bases 3-6 since they are in the active-site pocket of TREX1. In the TREX1-DNA simulation, the significant hydrogen bonding (occupancy >0.1) of the substrate with TREX1 includes b6-(O3′,O4′,O1P) with Glu20-OE1, Ala-N, Tyr129-OH, Ser178-OG, and His195-ND1; b5-(O4′,O2P) with Asn125-ND2 and Ser178-OG; b4-(N3,O2P) with Asn125-ND2, Ser176-OG, and Tyr177-N; b2-(N3,N4) with Ser173-O and Lys175-N; b1-(O5′,O1P) with Arg174-NH1. The polar atoms of DNA or RNA involved in the hydrogen binding with TREX1 are put in the parenthesis of a base. While b3 does not have any significant hydrogen bonding with TREX1 in the TREX1-DNA simulation, b3-(O2P,O2′,O4′) hydrogen bonds to Arg174-NH1 in the TREX1-RNA simulation, albeit the occupancy is not high and is mostly around 0.1. Overall, the pattern of hydrogen bonding in the TREX1-RNA simulation is similar to that in the TREX1-DNA simulation, but the occupancy values are much lower, indicating weaker hydrogen bonding interactions.

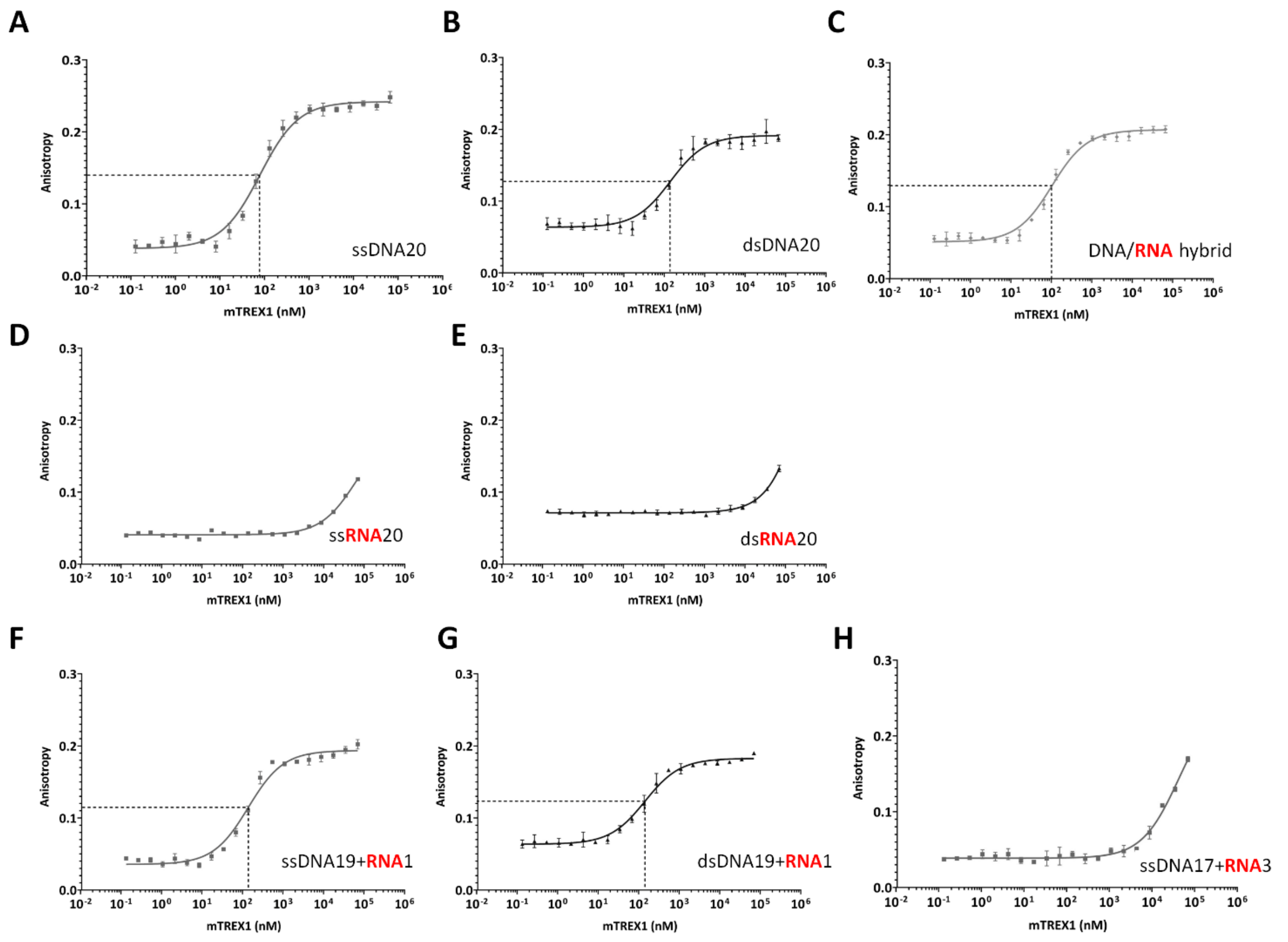

Supplementary Figure 12. Fluorescence anisotropy measurements of truncated mTREX1 on the various substrates. Fluorescence anisotropy of various nucleic acid substrates, including (A) ssDNA20 (ssDNA 20mer), (B) dsDNA (dsDNA 20 bp), (C) DNA/RNA hybrid (DNA/RNA hybrid 20 bp), (D) ssRNA20 (ssRNA 20mer), (E) dsRNA20 (dsRNA 20 bp), (F) ssDNA19+RNA1(ss19D1R; synthesized ssDNA 19 mer with additional one 3'-ended ribonucleotide), (G) dsDNA19+RNA1(ssDNA19+RNA1 anneals to ssDNA 20 mer) and (H) ssDNA17+RNA3 (ss17D3R; synthesized ssDNA 17 mer with additional three 3'-ended ribonucleotide). The substrates are labeled with FAM at the 5'-end is plotted as a function of added mTREX1 protein.

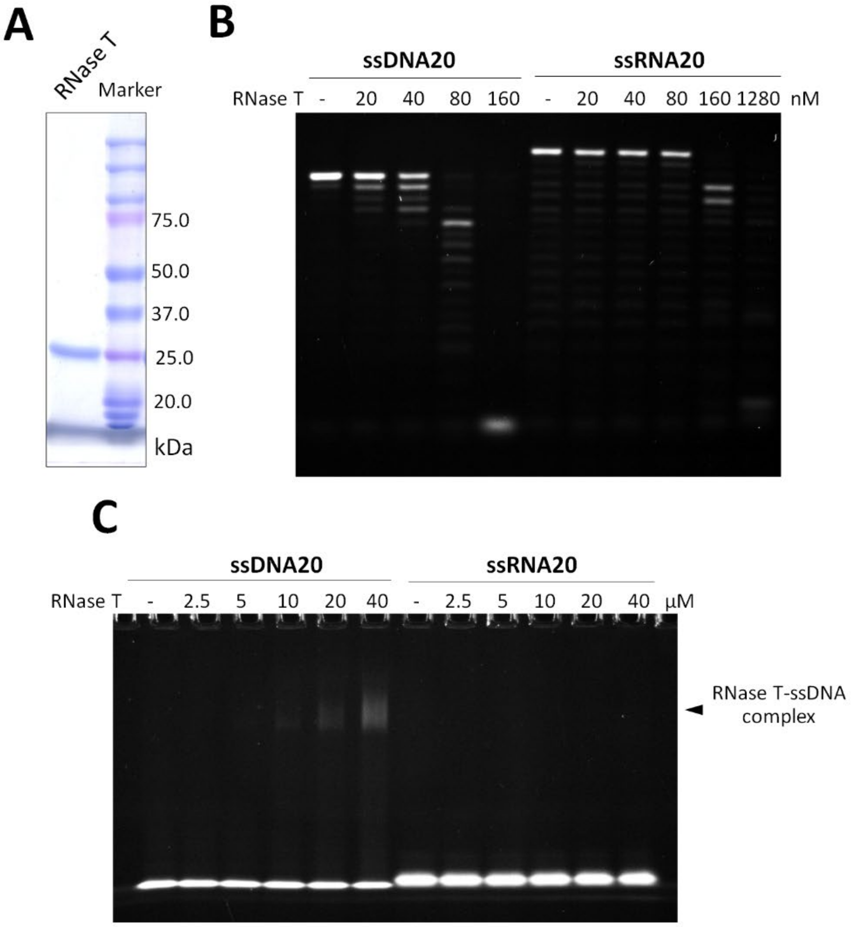

D

		AMP	dAMP
+ 2 mM $MgCl_2$	K_d (μM)	152.4 ± 10.54	179.9 ± 11.39
	R squared	0.9881	0.9902
No $MgCl_2$	K_d (μM)	140.6 ± 8.178	185.5 ± 11.35
	R squared	0.9912	0.9910

Supplementary Figure 13. The biochemical assays for RNase T in distinguishing DNA and RNA substrates (A) SDS-PAGE analysis of purified RNase T. (B)(C) Nuclease activity assays and Electrophoretic Mobility Shift Assay (EMSA) of RNase T against ssDNA and ssRNA substrates. The detailed process of EMSA is shown in the Supplementary Methods. The result shows RNase T digests ssDNA with higher efficiency than digests ssRNA. The substrate binding ability of RNase T on ssDNA is also higher than on ssRNA because the RNase T-substrate complex band formation was observed only when using ssDNA as substrate. (D) The Intrinsic Tryptophan Fluorescence (ITF) measurement of the K_d between RNase T and various nucleotides, AMP and dAMP, in the presence or absence of 2 mM $MgCl_2$.

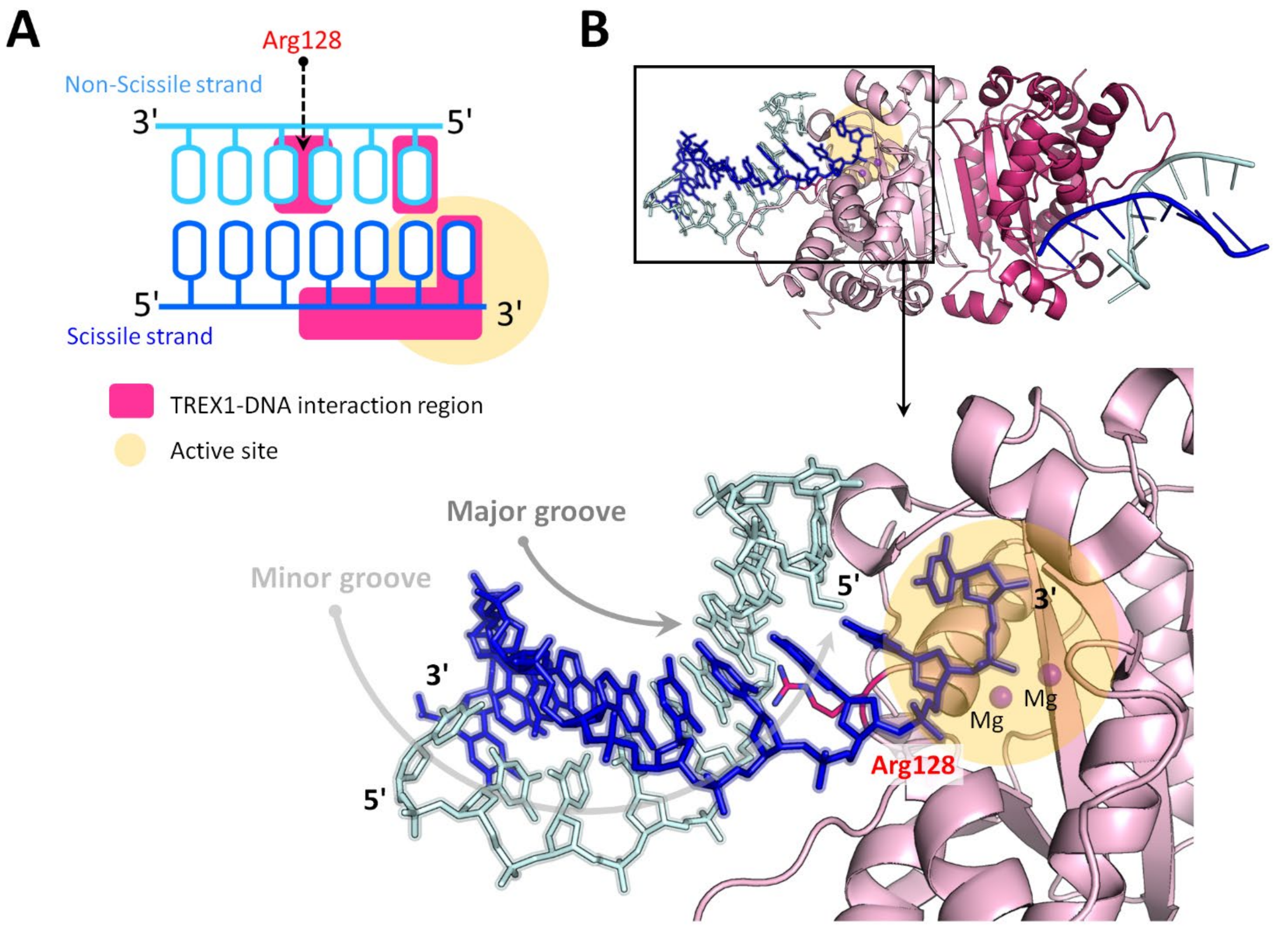

Supplementary Figure 14. Structural analysis of mTREX1 in complex with duplex DNAs. (A) Schematic of the interactions between mTREX1 and duplex DNA, which is based on three structures (PDB code: 5YWS, 5YWT, and 5YWU). (B) The structure of mTREX1-Y-structural DNA complex (PDB code: 5YWS). The Arg128 is inserted into the minor groove of Y-structural DNA. The scissile and non-scissile strands of dsDNA are colored blue and light blue.
